# Conformality of atomic/molecular layer deposited lithium terephthalate thin films

**DOI:** 10.1039/d6nr01994c

**Published:** 2026-09-08

**Authors:** Anish Philip, Milad Madadi, Joakim S. Jestilä, Antti J. Karttunen, Jussi Kinnunen, Maarit Karppinen

**Affiliations:** a Department of Chemistry and Materials Science, Aalto University FI-00076 Espoo Finland anish.philip@aalto.fi maarit.karppinen@aalto.fi; b Chipmetrics Ltd 80130 Joensuu Finland

## Abstract

The atomic/molecular layer deposition (ALD/MLD) technique is believed to provide unparallelled benefits for the fabrication of thin-film components for flexible 3D lithium-ion microbatteries, for which the nanoscale conformality of the active battery material layers is a critical requirement. For such an ALD/MLD-fabricated microbattery assembly, lithium-terephthalate (Li-TPA) is a prominent anode material candidate. Here we demonstrate the role of the lithium precursor in controlling the conformality of Li-TPA films. We first develop two new ALD/MLD processes for Li-TPA by pairing the Li precursors, lithium *tert*-butoxide (LiO^*t*^Bu) and lithium bis(trimethylsilyl)amide (Li-HMDS) with terephthalic acid (TPA); in previous studies, Li-TPA thin films have been grown from lithium 2,2,6,6-tetramethyl-3,5-heptanedionate (Li-THD). Then, using state-of-the-art lateral high-aspect-ratio (LHAR) test structures, we evaluate the three ALD/MLD processes: LiO^*t*^Bu + TPA, Li-HMDS + TPA and Li-THD + TPA, for both the essentially constant film-growth range (conformality) and the ultimate extent of the film growth (penetration depth) along the LHAR cavities; these parameters are determined using a novel imaging ellipsometry approach in addition to scanning electron microscopy characterization. The results highlight the importance of the size and nature of the lithium precursor. Notably, among the three Li precursors, Li-HMDS yielded Li-TPA films of superior conformality and penetration depth characteristics. Through a detailed computational investigation of the precursors, including conformer screening, DFT optimizations and accurate DLPNO-CCSD(T) energy calculations, we correlate the experimentally observed trends with the most likely oligomeric forms of the precursors at reactor conditions.

## Abstract 2

Conformality of thin films for 3D lithium-ion microbatteries deposited by atomic/molecular layer deposition (ALD/MLD) depends on the propensity of the precursors to form oligomers.

## Graphical abstract

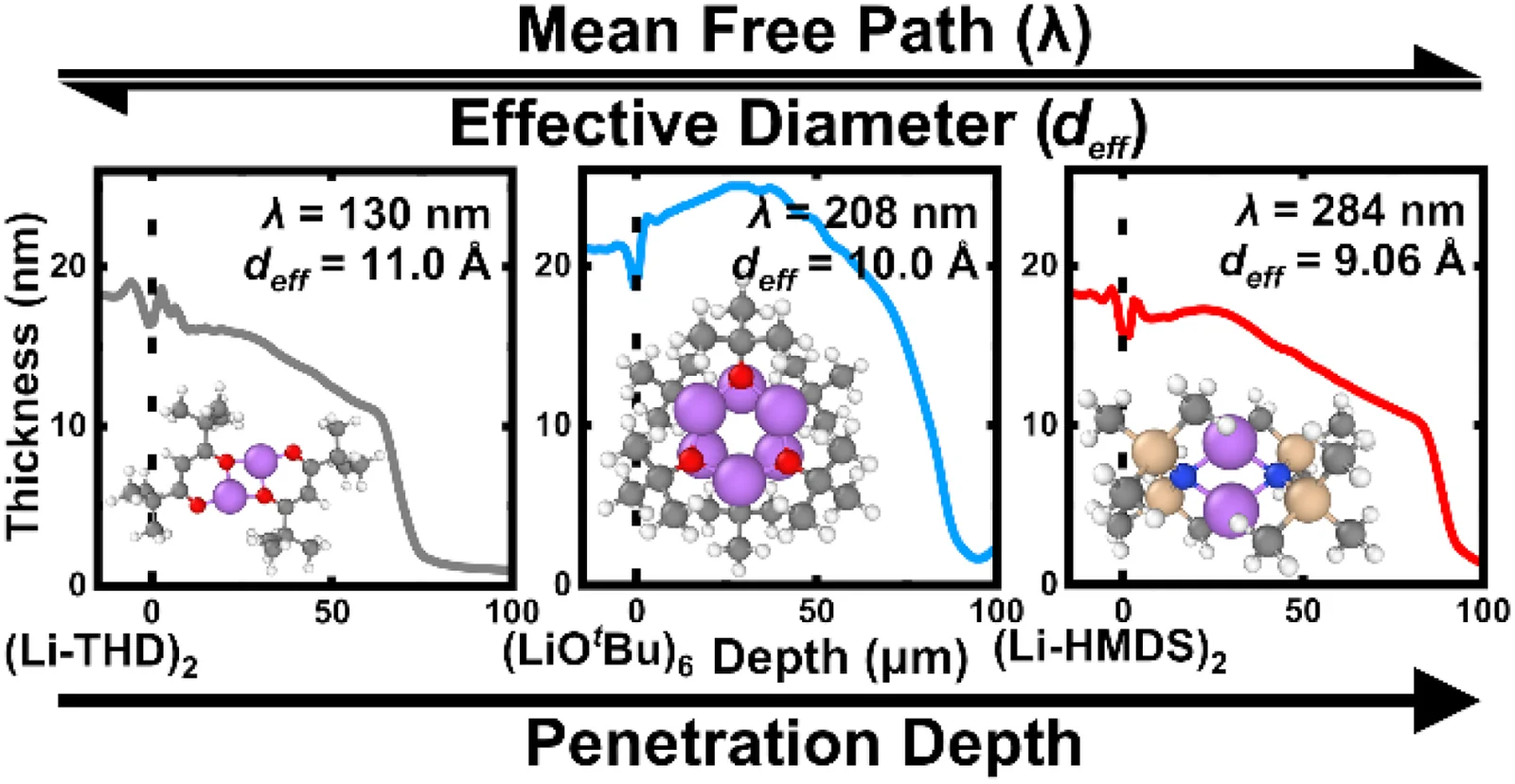

## Introduction

The continuous progress in the miniaturization of electronic devices necessitates the development of compact energy storage devices, *i.e.*, thin-film microbatteries.^1–5^ A compelling way to fabricate electrodes for microbatteries is the use of atomic layer deposition (ALD) which – in principle – enables pinhole-free thin films with an unparalleled degree of conformality even on challenging 3D surfaces with high aspect ratios.^3,6–12^ Adding organic moieties to the process through molecular layer deposition (MLD)^13–15^ vastly increases the number of possible depositable structures and achievable material properties, *e.g.*, electrodes with tunable redox properties and superior mechanical flexibility.^16–18^ Replacing conventional toxic and less abundant inorganic electrode materials with organics is also perfectly in line with today's sustainability requirements.^19–21^

Lithium terephthalate (Li-TPA) is an excellent example of promising ALD/MLD-grown electrode materials; this crystalline lithium–organic material possesses the required redox chemistry and electrical properties for its use as a negative electrode.^22,23^ The recent progress in ALD/MLD battery material research has indeed demonstrated the realization of an all-ALD/MLD-fabricated thin-film microbattery with Li-TPA as the working anode material.^19–21,24^

Although thin-film microbatteries possess many positive features essential for advanced battery solutions, there are still challenges that need to be addressed for them to be used in real-life applications. One of the major challenges with these batteries is their inferior energy density,^25^ as thin-film electrodes inherently have a minuscule volume and consequently a small charge storage capacity. Ultra-thin electrodes, on the other hand, are superior in power density, *i.e.*, the charging/discharging speed. An evident solution to optimize the performance of thin-film batteries is to deposit ultra-thin active material layers on high-aspect-ratio (HAR) surfaces to significantly increase the effective surface area^26^ and active-material volume, such that the energy density can be increased without compromising the high power density structuring^27–29^ For proper electrochemical performance, the active material layers in the 3D thin-film batteries need to be of constant thickness over the entire microbattery device.^12,30^ Moreover, the HAR cavities should be relatively deep to create large enough surface areas. Hence, both the degree of conformality and the absolute penetration depth (PD) are important parameters.

In this study, using state-of-the-art lateral high-aspect-ratio (LHAR) test structures and characterization techniques including a novel imaging ellipsometry approach, we quantitatively assess the conformality and penetration depth performances of Li-TPA thin films fabricated *via* ALD/MLD from three different lithium precursors. The results clearly demonstrate the influence of the precursor bulkiness in controlling both the degree of conformality and the ultimate depth of film penetration into the high-aspect-ratio cavities. To support the experimental findings, we will show through quantum chemical calculations that the lithium precursors investigated have different aggregation tendencies in the gas phase, leading to various oligomeric structures with different mean free paths, influencing the observed conformality behaviour. We believe the results and deeper understanding gained here for the conformality and film coverage characteristics of Li-TPA thin films utilizing imaging ellipsometry in combination with quantum chemical calculations present an important model case of efficient precursor screening for an industry relevant ALD/MLD process.

## Experimental

**Fig. 1:**
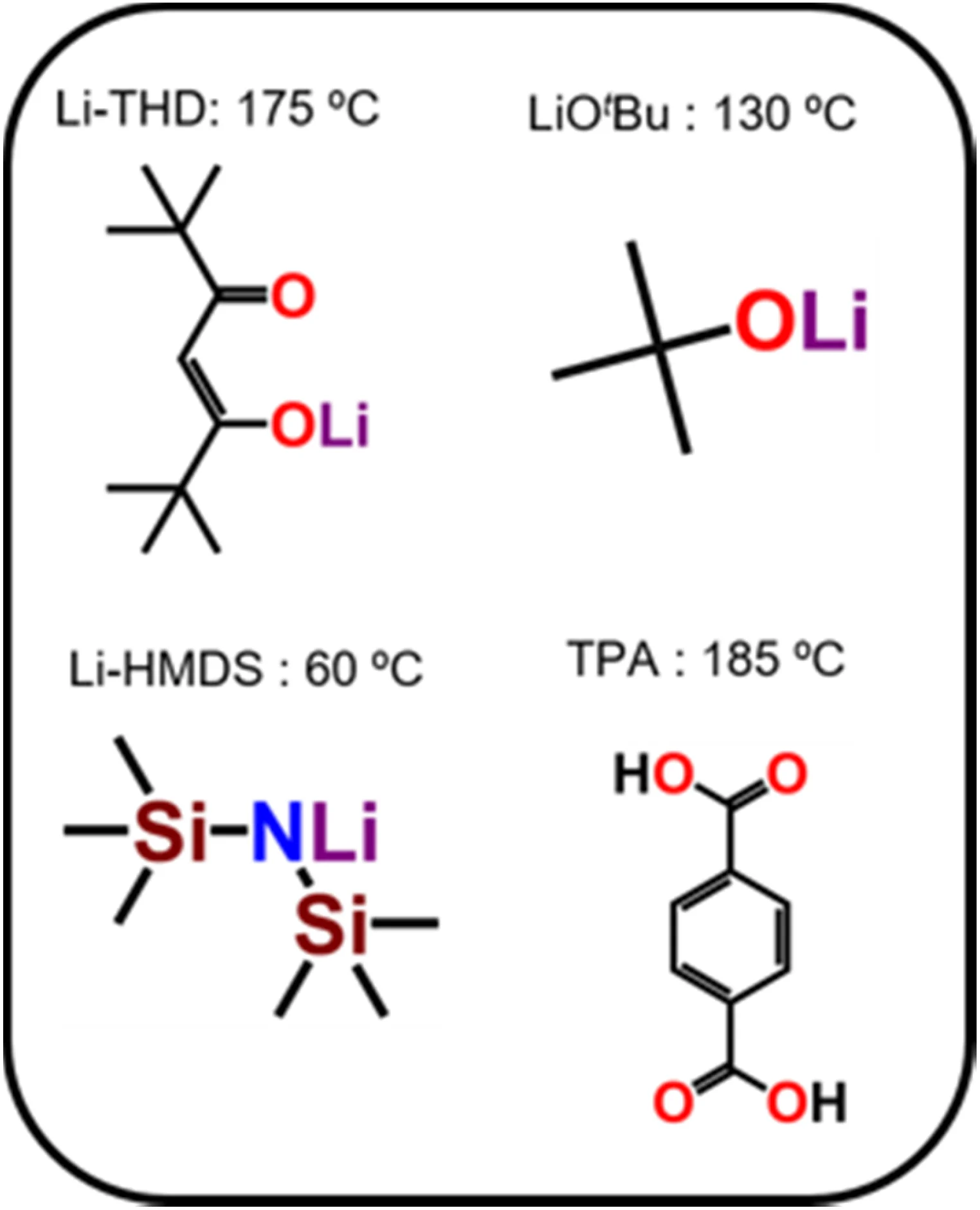
Monomeric molecular structures of the lithium precursors and the organic precursor used in this study; the heating temperatures used for their vaporization are also indicated.

The Li-TPA thin films were grown from three different solid lithium precursors: lithium *tert*-butoxide (LiO^*t*^Bu, Alfa Aesar, 99%), lithium bis(trimethylsilyl)amide (Li-HMDS, Sigma-Aldrich, 97%) and Li-THD (THD = 2,2,6,6-tetramethyl-3,5-heptanedionate), in combination with terephthalic acid (TPA; Tokyo Chemical Industry CO., Ltd, >99.0%); the monomeric molecular structures of the precursors are shown in Fig. 1. For the thin-film depositions, both the Li precursor and the organic TPA precursor were placed in open boats inside the reactor (flow-type hot-wall ALD reactor; F-120 by ASM Microchemistry Ltd) where they were heated for vaporization (TPA: 185 °C, LiO^*t*^Bu: 130 °C, Li-HMDS: 60 °C, Li-THD: 175 °C); the temperatures used were chosen based on our previous experience using these precursors in other ALD and MLD processes.^23,31^ All depositions were first carried out on 2.0 × 2.0 cm^2^ Si(100) substrates (Okmetic Oy). Nitrogen (>99.999%, Schmidlin UHPN 3000 N_2_ generator) was used as a carrier and purging gas, and the pressure inside the reactor was maintained between 5–7 mbar.

For confirming the completion of the expected reaction and identifying the types of chemical bonding involved in the Li-TPA thin films, both Fourier transform infrared (FTIR; Bruker ALPHA II) and Raman spectroscopy (Renishaw inVia Qontor; 532 nm) measurements were carried out. The FTIR measurements were performed in transmission mode in the wavenumber range of 400–4000 cm^−1^. The resolution was 4 cm^−1^, and each given spectrum is an average of 100 measured spectra. For removing the interference from silicon, a blank Si spectrum was subtracted from the thin-film spectrum. The Raman measurements were performed with a laser power of 10% and an accumulation time of 30 s, and each given spectrum is an average of 30 measured spectra.

The film thickness and optical properties were characterised using a spectroscopic ellipsometer (SE; HORIBA France SAS UVISELPLUS), operating within an energy range of 0.6–6.5 eV and an incidence angle of 70°. Data modelling was carried out using the accompanying software and the included references. The optical model was constructed using a silicon reference as the substrate layer, followed by a native 2 nm SiO_2_ layer. For the data fitting, Sellmeier dispersion model was used. The quality of fitting was confirmed with a lower *χ*^2^ value. A representative fit for all three precursors with 50 ALD/MLD cycles are given in Fig. S1 (SI). X-ray reflectivity (XRR; Rigaku SmartLab diffractometer; Cu Kα radiation, *λ* = 1.5418 Å, HyPix-3000 detector, and Ge (220) × 2 double-bounce monochromator) measurements were carried out to confirm the film thickness especially for thinner films. To investigate the crystallinity of thinnest films, grazing-incidence X-ray diffraction (GIXRD) measurements using the same Rigaku SmartLab diffractometer were performed at an incidence angle of 0.5°. The growth-per-cycle (GPC) values reported were calculated from the thickness values obtained from the SE data. The crystallinity/structural details of the films was addressed based on X-ray diffraction data (XRD); Bruker D8 Advance Vario 1 two-circle diffractometer (*θ*–2*θ* Bragg–Brentano mode; Cu Kα1 radiation (*λ* = 1.5406 Å); Ge (111) monochromator (Johansson type); Lynx Eye detector). The diffraction patterns were collected in the 2*θ* range of 10–50°.

Scanning electron microscopy (SEM; JEOL JSM-IT800HL) was used for examining both the surface morphology and the penetration depth of the Li-TPA films inside the LHAR cavity. Atomic force microscopy (AFM; Bruker Dimension Icon) measurements were performed for the planar samples grown silicon substrate; the measurements were conducted in the peak force tapping mode at 218 kHz (frequency) and at 1 Hz (line rate). The AFM images were then analyzed with the NanoScope Analysis 3.00 software.

For the conformality studies, the depositions were performed on PillarHall® LHAR test structures (LHAR5, Chipmetrics Ltd). The gap height (*i.e.*, pore diameter) of the LHAR test chips was 500 nm. The top-roof polysilicon membrane from the LHAR structure was removed with an adhesion tape after the film deposition.^31–33^ A small piece of adhesion tape was gently glued on the chip surface and then pulled away to peel off the polysilicon membrane from that side, thereby exposing the film for analysis (Fig. S2, SI). Then, any analytical technique with sufficient spatial resolution compatible with planar materials can assess the conformality of the film growth. In this study, we used SEM for imaging the film grown on the LHAR substrate and for measuring the penetration depth. An imaging ellipsometer (EP4-300, Park Systems) was used for the accurate assessment of conformality. The Sellmeier dispersion model was used as a simple stack with fixed native SiO_2_ on top of single-crystal silicon substrate; a detailed description is given in SI. The thickness profile (Fig. S2d, SI) was obtained from the film to identify the thickness changes along the film penetration and to compare the film thickness inside the cavity to the thickness at the opening area, where the growth behaviour is similar to that on planar silicon.

## Computational

Experimental results of several previous studies have revealed that the lithium precursors employed here may not be monomers in the gas phase, in particular under ALD-reactor conditions.^34–36^ For the three precursors studied, previous studies suggested hexameric LiO^*t*^Bu and tetrameric Li-THD and Li-HMDS.^35–38^ To find the most prevalent oligomeric species (monomer, dimer, trimer, tetramer, pentamer or hexamer) for each of the lithium precursors, we first determined the most stable conformations using the conformer-rotamer ensemble sampling tool with default settings.^39,40^ The lowest-energy conformations of each oligomer (Fig. S3, SI) were subsequently relaxed using B3LYP-D4/def2-TZVPP level of theory,^41–46^ and were confirmed to be true local minima through frequency calculations (Table S1), which also provided the thermal corrections to the Gibbs free energies. The electronic total energies of the resulting structures were then recomputed with the DLPNO-CCSD(T) method (Table S2),^47,48^ including extrapolation to the complete basis set limit (using the def2-TZVPP/def2-QZVPP basis sets) to minimize any errors due to basis sets on the cluster binding energies, as this has been shown in previous studies to be critical for obtaining accurate equilibrium constants for dimerization, and by extension, for oligomerization in general.^34^ The DFT and coupled-cluster computations, along with the basis set extrapolations, were done with ORCA 6.1.1^48–51^ employing the default *normalPNO* settings for DLPNO-CCSD(T). We made use of the RI approximation to speed up the SCF-part of these computations.^52^

Furthermore, we estimated the mean free path (*λ*) for the most prevalent oligomeric species to help rationalize the experimental observations. An effective diameter (*d*_eff_) based on the computed rotational constants was first computed, to account for the different shapes of the oligomers, as simple geometric parameters might lead to misleading results, especially when considering a mixture of spherical and elongated, rod-like geometries. The effective diameter was estimated as two times the van der Waals corrected radius of gyration (+1.2 Å per H atom), the latter being calculated by using the moments of inertia derived from the computed rotational constants. Finally, the mean free paths were calculated using an analogous method,^53^ but with the effective diameters being used in place of geometric diameters in the calculation of collision cross sections. Full details of calculations are provided in the SI (Tables S1–S22). Images of the molecular structures of the oligomers were rendered in OVITO.^54^

## Results and discussion

### ALD/MLD processes of Li-TPA on planar substrates

**Fig. 2:**
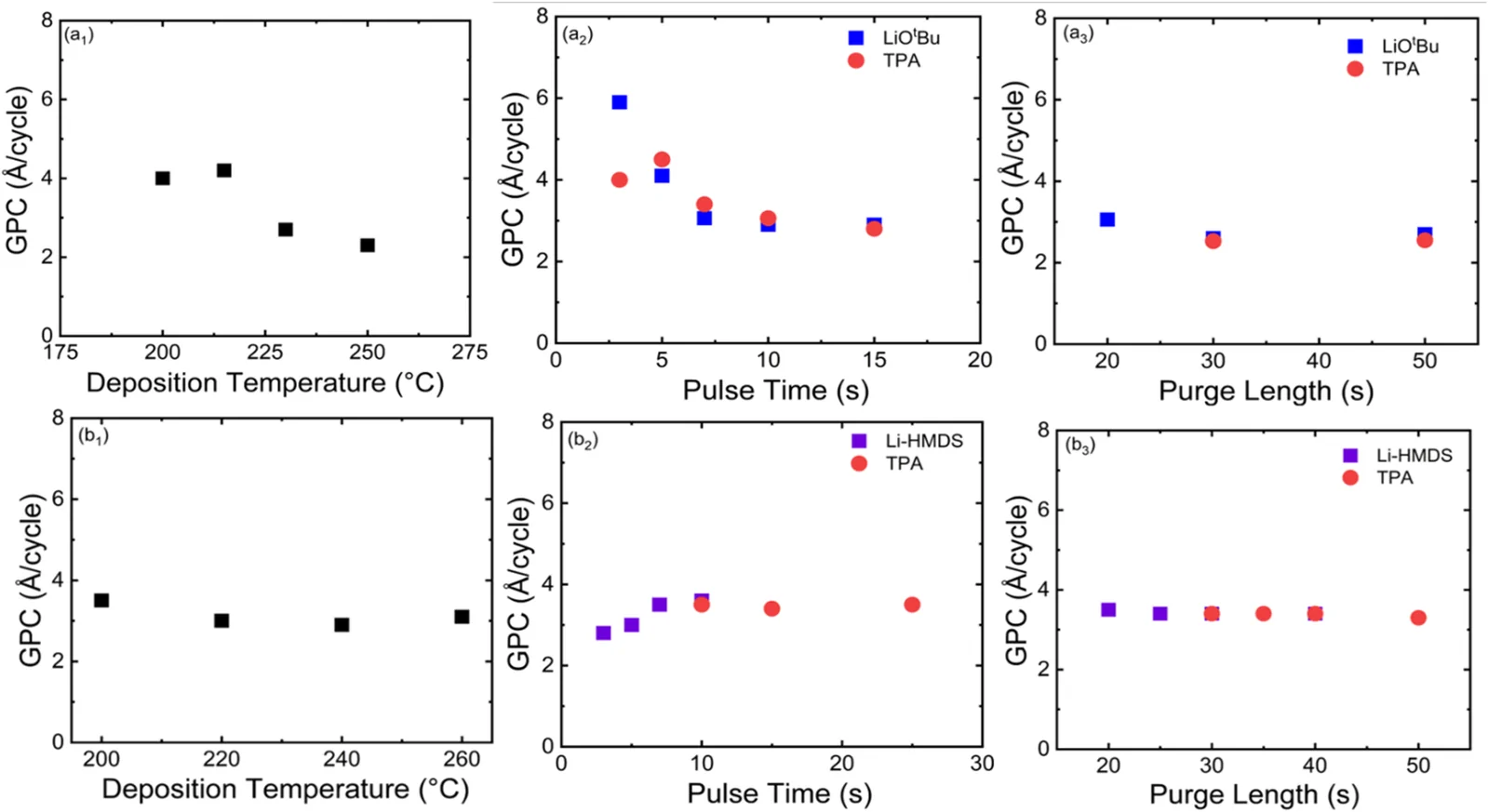
Optimization of the two ALD/MLD processes developed in this study: (a) LiO^*t*^Bu + TPA, and (b) Li-HMDS + TPA. GPC *versus* deposition temperature studied with the pulse/purge sequences of (a_1_) 5 s LiO^*t*^Bu/5 s N_2_/10 s TPA/30 s N_2_, and (b_1_) 5 s Li-HMDS/5 s N_2_/10 s TPA/30 s N_2_. Saturation of GPC (a_2_ & b_2_) with individually increased precursor pulse lengths, and (a_3_ & b_3_) individually increased purge length after the precursor pulses.

**Fig. 3:**
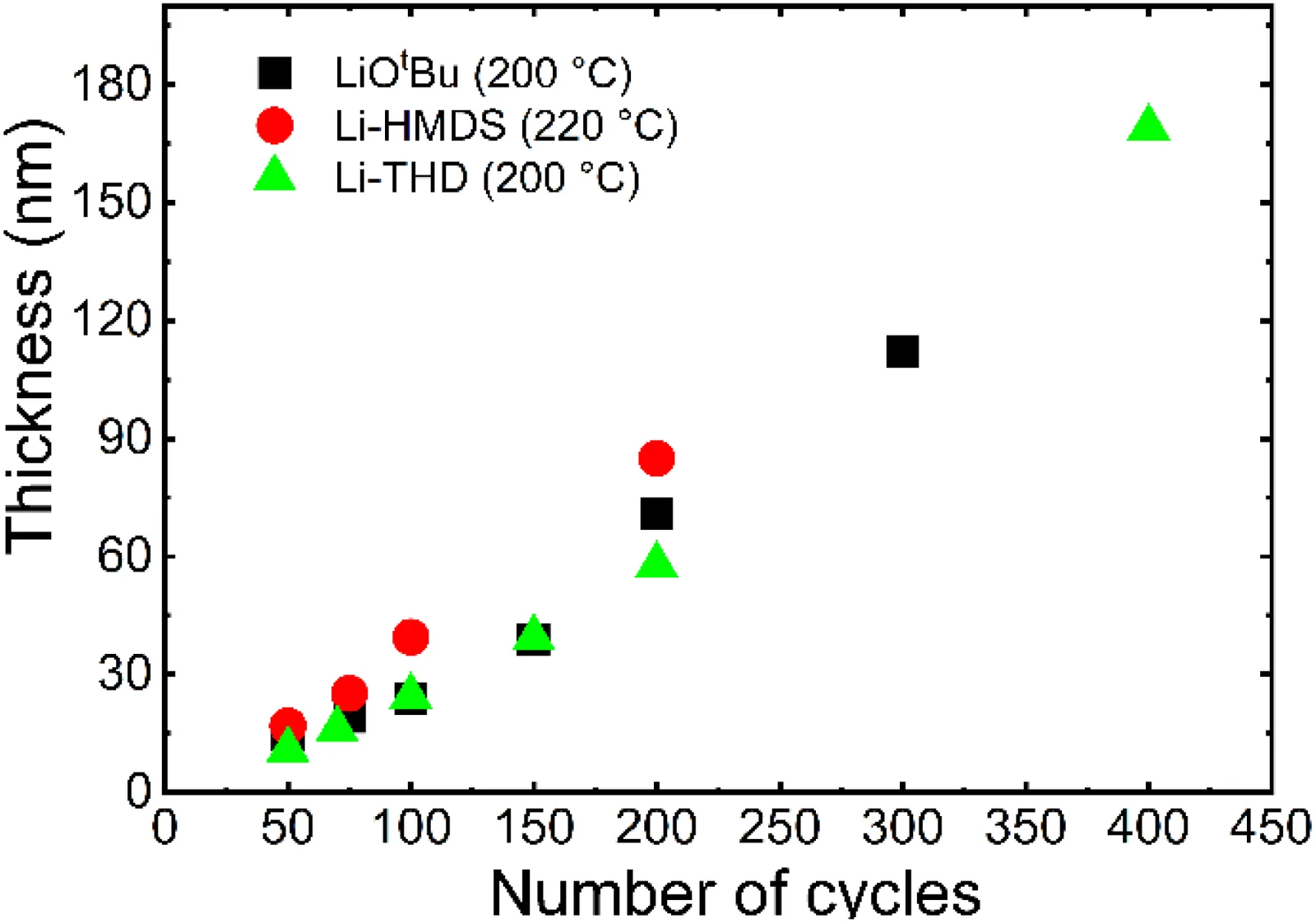
Film thickness as a function of the number of ALD/MLD cycles applied for the three Li-TPA processes studied; the data for the Li-THD + TPA process were taken from our previous publication.^23^ The optimized deposition temperatures used are indicated in parentheses.

Our original ALD/MLD process for the deposition of *in situ* crystalline Li-TPA thin films was based on Li-THD as the lithium precursor.^23^ Thus, we started the present study by searching for the optimal process parameters for the two Li-TPA processes not previously reported, *i.e.*, LiO^*t*^Bu + TPA and Li-HMDS + TPA. These experiments were carried out using standard planar silicon substrates; the results are summarized in Fig. 2 and 3.

For both the new processes, the GPC value slightly decreases with increasing deposition temperature, as typically seen for many ALD/MLD processes (Fig. 2a_1_ and b_1_). For the rest of the experiments, we fixed the deposition temperature to 200 and 220 °C for the LiO^*t*^Bu and Li-HMDS based processes, respectively, to maximize the GPC value. Here it should be mentioned that for LiO^*t*^Bu, two temperature ranges with different saturated GPC values were observed; we selected the lower deposition temperature (200 °C) because it yielded the higher GPC value, noting that this chosen temperature was not very far from the deposition temperature used for the other two Li-TPA processes studied. Then, regarding the precursor pulse and purge lengths, the surface saturation conditions were achieved with the following pulse/purge sequences: 7 s LiO^*t*^Bu/30 s N_2_/10 s TPA/30 s N_2_, and 7 s Li-HMDS/25 s N_2_/10 s TPA/35 s N_2_. In case of LiO^*t*^Bu, the GPC value initially decreases with an increase in the pulse lengths of the precursors, which is opposite to what is generally expected. We tentatively believe this is due to the hygroscopic nature of LiO^*t*^Bu.^36^ Moreover, the concomitant change in the refractive index of the films (Fig. S4, SI) suggests that also the resultant material characteristics may change depending on the precursor pulsing times.

Finally, in Fig. 3 the film thickness is plotted against the number of ALD/MLD cycles applied for the two new processes, and also for our previously reported Li-THD + TPA process for comparison.^23^ Similarly to the Li-THD + TPA case,^23^ both the Li-HMDS + TPA and LiO^*t*^Bu + TPA processes showed an essentially linear film growth behaviour with the lower cycle numbers, while at the higher cycle numbers, the GPC gradually increased. This slightly enhanced film growth at the higher cycle numbers could be attributed to several factors including the increase in film roughness or possible interaction of precursors with moisture which is very common for alkali metal precursors.^36^

### Basic characterization of Li-TPA thin films

**Fig. 4:**
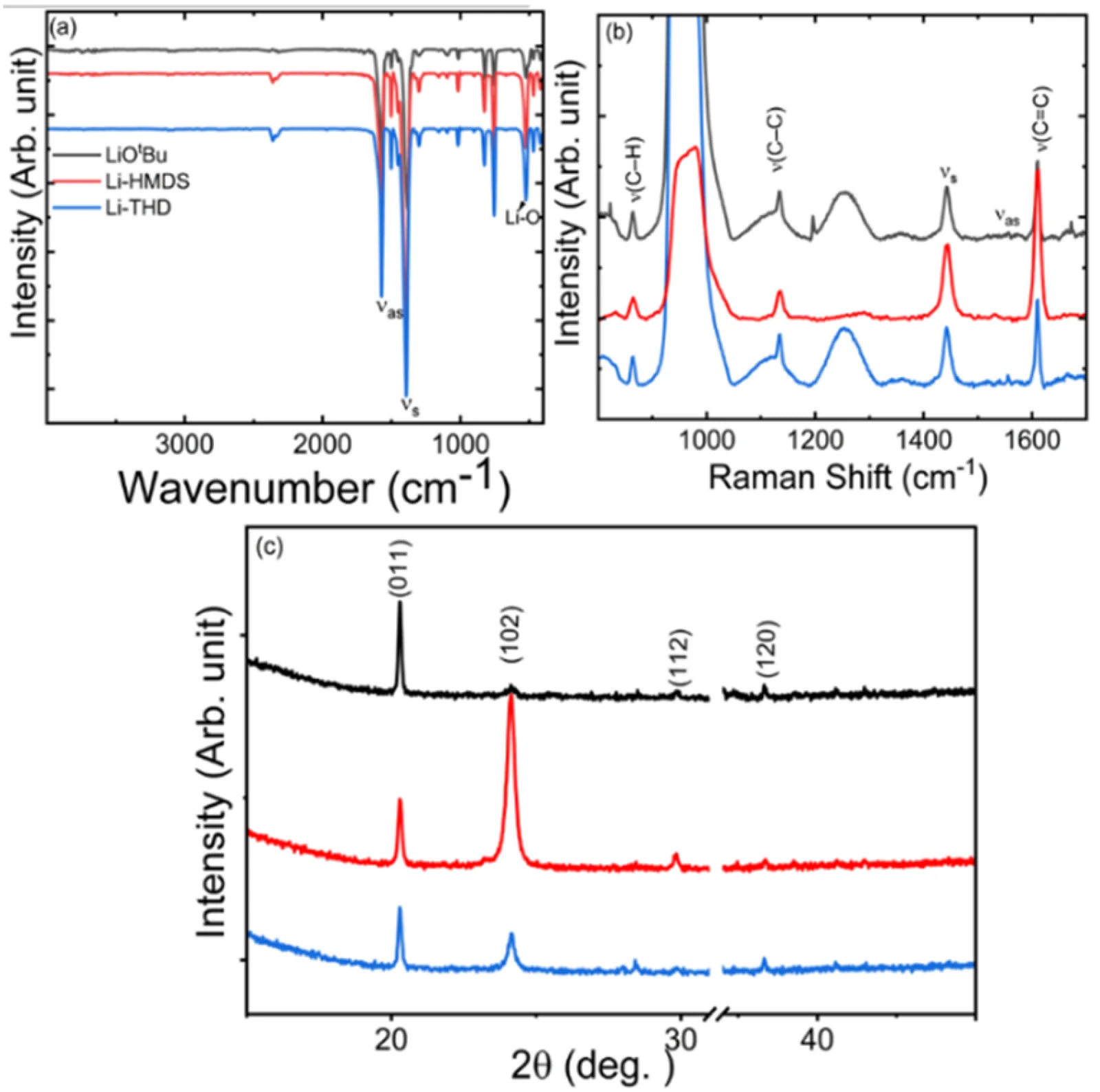
Chemical and structural characterization data for Li-TPA thin films deposited with 400 ALD/MLD cycles on planar silicon substrates: (a) FTIR spectra, (b) Raman spectra, and (c) XRD patterns.

According to the FTIR spectra (Fig. 4a), both the LiO^*t*^Bu + TPA and Li-HMDS + TPA processes result in Li-TPA thin films, exhibiting spectral features identical to those previously seen for the Li-THD + TPA process.^23^ The absence of the characteristic band corresponding to the –OH stretching around 2500–3000 cm^−1^ indicates that the co-reactant TPA is completely consumed during the ALD/MLD process, leaving no residues in the film. The spectra moreover verify the presence of expected terephthalate group in the Li-TPA films with the well-known carboxylate-group fingerprint, *i.e.*, symmetric (*ν*_s_) and asymmetric (*ν*_as_) stretching bands at 1394 and 1572 cm^−1^, respectively. For all the three processes (Fig. S5, SI), the peak intensity was found to increase linearly with the number of ALD/MLD cycles applied, as expected for an ideal ALD/MLD process. The bridging mode of TPA binding to the lithium atoms was confirmed^55^ from the magnitude of splitting between these bands, *i.e.*, 178 (=1572–1394) cm^−1^ for all the samples irrespective of the lithium precursor.

Raman spectroscopy measurements were used to further verify the Li-TPA deposition; in the Raman spectra (Fig. 4b), the characteristic peaks^56^ corresponding to the aromatic ring and the binding between the lithium atom and TPA moiety are clearly visible. The intense peak observed at 1612 cm^−1^ corresponding to C=C stretch affirms the aromatic backbone in the structure. The other visible Raman peaks observed at 864 and 1136 cm^−1^ were assigned to the C–H out-of-plane bending from the aromatic backbone, and C–C bond stretching between the aromatic ring and the carboxylate group. The symmetric (*ν*_s_) and asymmetric (*ν*_as_) stretching peaks of the COO– group were seen at 1445 and 1556 cm^−1^, respectively. At higher cycle numbers, the Raman peak corresponding to *ν*_as_ stretching got weakened or was not seen at all, while it was moderately strong in the thinner films. The *ν*_as_ peak is visible for all the three Li-TPA samples deposited with 100 cycles (Fig. S6, SI). The reason behind the observed trend could be enhanced crystallinity with increasing film thickness. For the very thin and less crystalline films the asymmetric stretching vibration could become more dominant. The GIXRD patterns for the films grown with 100 cycles (Fig. S6, SI), *i.e.*, in the initial stages of film growth, were very similar for all the three precursor cases.

The XRD data confirmed that the films were highly crystalline as expected (Fig. 4c). Interestingly, the precursor had an influence on the orientation; while the Li-TPA films deposited from LiO^*t*^Bu exhibited enhanced (011) peak intensity, those deposited with Li-HMDS showed rather an increased (102) peak intensity. The films deposited from Li-THD did not show any specific orientation, in line with previous reports.

**Fig. 5:**
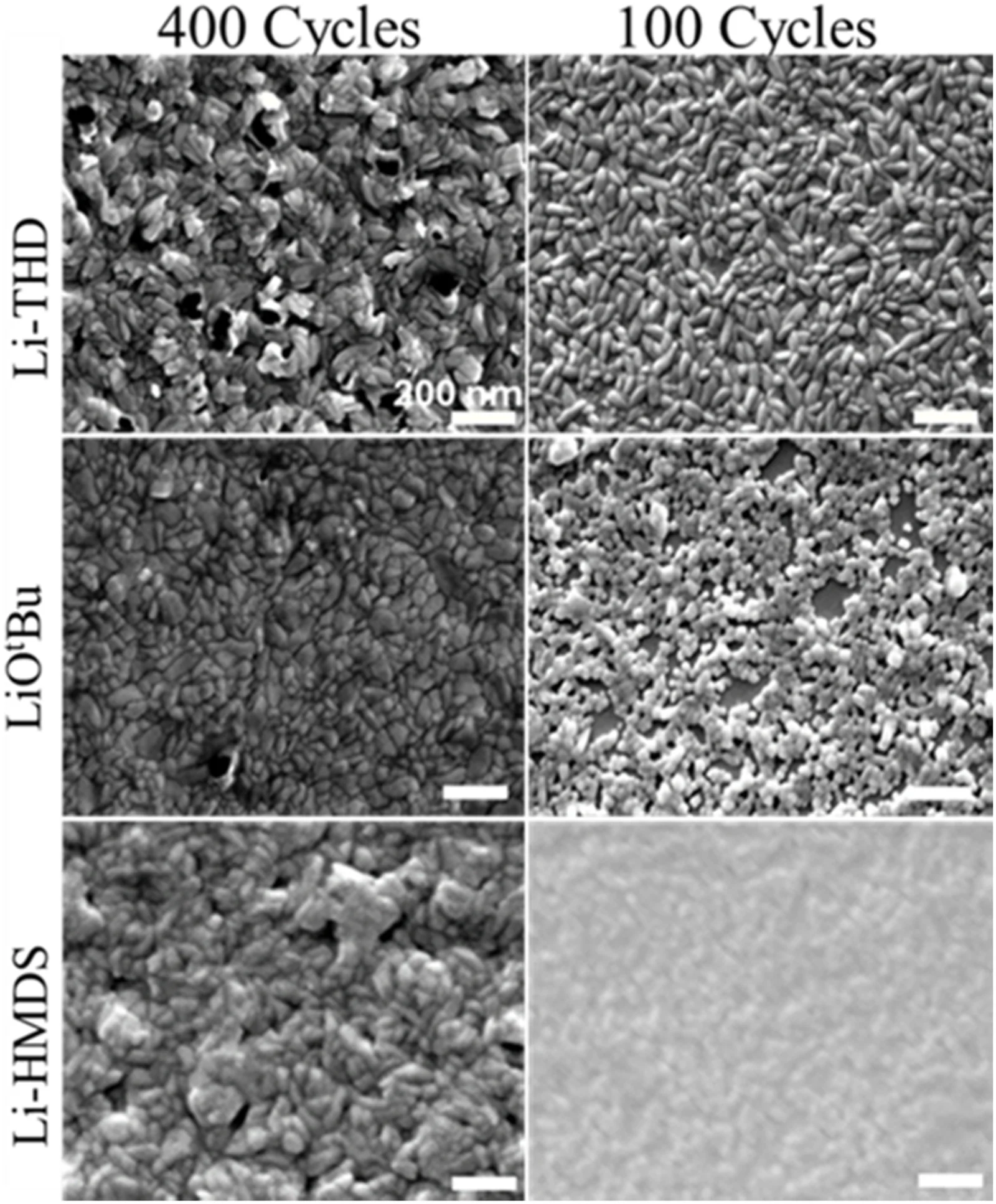
SEM images of the grain structure of thicker and thinner Li-TPA films (400 and 100 cycles). The lithium precursor used is indicated on the left-hand side of the images. The scale bar in each image is 200 nm.

**Fig. 6:**
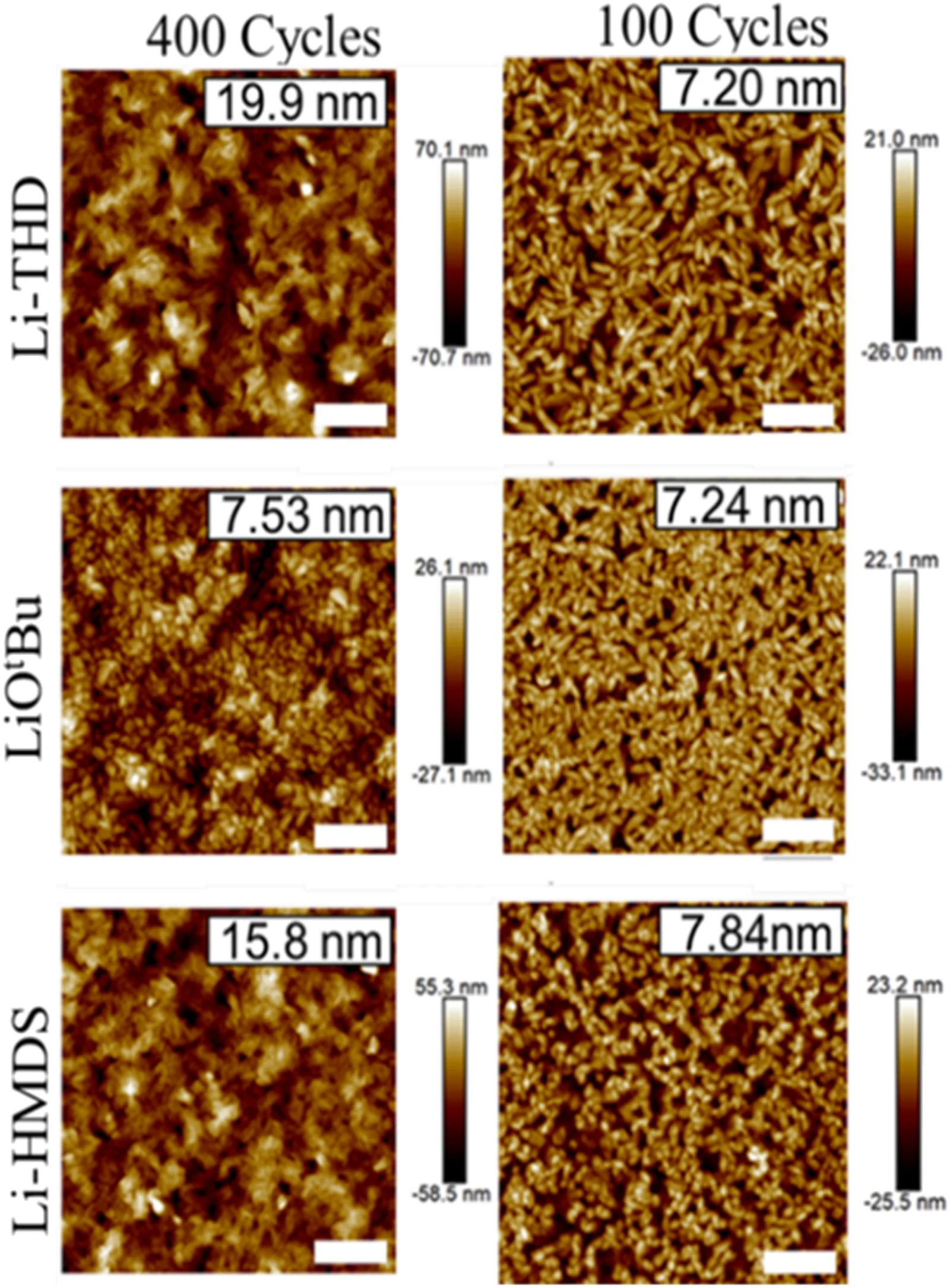
AFM images of the grain structure of thicker and thinner Li-TPA films (400 and 100 cycles). The lithium precursor used for Li-TPA deposition is indicated on the left-hand side of the images. The RMS roughness of the films (in nm) is indicated on the images. The scale bar in each image is 400 nm.

The morphology of the Li-TPA thin films was investigated using both SEM (Fig. 5) and AFM (Fig. 6) techniques. After the first deposition cycles, the grains were found to be more granular with better dispersity in the case of the Li-THD + TPA and Li-HMDS + TPA processes, while for the LiO^*t*^Bu + TPA process, the grains were differently shaped and more agglomerated with poorer dispersity and film discontinuity. We tentatively attribute these observations to the different oligomers formed by the three Li precursors: as discussed below based on our computational study, LiO^*t*^Bu tends to form hexamers under the deposition conditions used, while the other two Li precursors tend to form dimers. As in previous reports,^57^ with increasing number of deposition cycles, the grain coalescence was enhanced, resulting in improved film continuity. In all three cases, the films appeared continuous after 250 ALD/MLD cycles at the latest (Fig. S7, SI).

As expected, the thinner films were comparatively smoother. In all three cases, the RMS roughness was between 7–8 nm for the 100-cycle films. In the case of LiO^*t*^Bu, the RMS roughness remained low (7.5 nm) also for the thicker (400 cycles) films, while for Li-THD (19.9 nm) and Li-HMDS (15.8 nm) the roughness was found to increase with increasing film thickness. According to the XRD data, Li-TPA films deposited from LiO^*t*^Bu exhibited enhanced orientation along the (011) plane and that may have attributed to the observed smoothness for the thicker films deposited using this precursor.

Finally, we investigated the long-term stability of our Li-TPA thin films; this is particularly important considering their potential use as anode materials in microbattery applications. Impressively, FTIR spectra collected for the films after storing them in ambient conditions for one and half years were essentially identical to those collected for the same films immediately after the deposition (Fig. S8, SI); the minor feature appearing in the 3600–3900 cm^−1^ is presumably due to CO_2_ adsorption on the film surface during the aging.^58^ No significant changes were seen in film thickness after this aging either, further corroborating their observed stability.

### Conformality of Li-TPA films on LHAR substrates

The conformality characteristics of our Li-TPA films were examined using LHAR test chips. Unlike conventional vertical high-aspect-ratio structures, these lateral chips enable conformality determination without cross-sectional analysis.^31,59^ For all three lithium precursors, the precursor/purge pulsing sequence used for most of the LHAR experiments was 7 s Li-precursor/50 s N_2_/10 s TPA/50 s N_2_. Here, the longer N_2_ purge pulses were employed in case to better ensure the excess precursors and biproduct molecules were efficiently purged out from the longer cavities so that any possible CVD type growth could be mitigated.

**Fig. 7:**
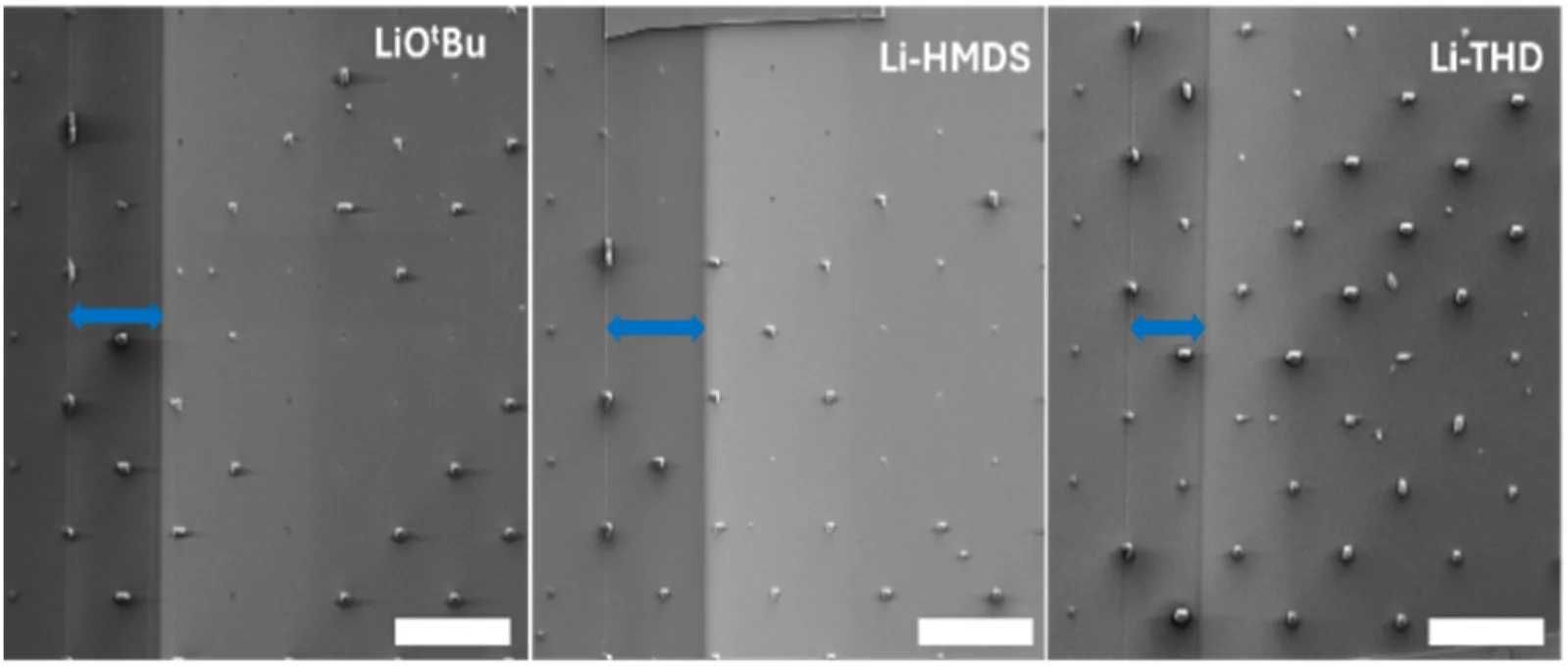
SEM images of Li-TPA thin films deposited with 50 cycles onto LHAR substrates using different Li precursors. Following pulsing sequence was used: 7 s Li-precursor/50 s N_2_/10 s TPA/50 s. In each image, the penetration depth is indicated with a blue double-sided arrow. The scale bar in each image is 100 µm. The lithium precursor used is indicated in each figure.

First, we used SEM analysis to estimate the film penetration for all the three processes (Fig. 7). An additional SEM image for the Li-THD + TPA process is given in the SI (Fig. S9) with a detailed explanation of the various investigated areas of the exposed surface investigated. The contrast difference at the borderline between the film and the uncoated cavity surface gives us a measure of how deep the film has grown inside the cavity.^31,60^ The results indicated that the growth of Li-TPA inside the cavity is highly lithium precursor dependent. For the Li-THD + TPA process, a relatively low PD value of 69 µm was obtained, whereas the processes with LiO^*t*^Bu (84 µm) and Li-HMDS (90 µm) resulted in clearly deeper film growth. Steric hindrance due to the large size of the dimeric Li-THD precursor could be reason for the comparatively lower PD value seen for the Li-THD + TPA process, limiting the ability of this precursor to proceed deep inside the trenches. The fact that among the LiO^*t*^Bu and Li-HMDS precursors a slightly shorter PD was observed for LiO^*t*^Bu could be understood by the stabilizing oligomer cluster formation which increases the overall size of LiO^*t*^Bu;^36^ the oligomer formation is typical for metal alkoxides in gas phase and will be discussed below in more detail based on our computational data. We also emphasize that, in the current contrast-based PD evaluation approach, only the darker-coloured region where the film is still clearly detectable was used to define the PD value. The lighter-coloured region that followed (representing an ultra-thin film or a second front)^60^ was not considered in the PD calculation.

Although the PD measurements from SEM images provide valuable information on how deeply the film grows within the cavity, they do not necessarily offer a full picture of the film's conformality, *i.e.*, the cavity deepness range within which the film grows at an essentially constant GPC rate. Thus, we employed imaging ellipsometry, which to the best of our knowledge has not been previously employed for conformality measurements, to accurately assess the degree of conformality of the present Li-TPA films. Most importantly, the used Sellmeier dispersion model showed an excellent fit with the measured data (Fig. S10, SI). The RMSE values (Fig. S11, SI) obtained from the fitting indicate that the fit for deposited film thickness is reliable (RMSE < 10) at the opening area (flat area on the left of membrane edge), at the middle conformal area and PD when the thickness is extremely small. The main differences in RMSE are observed at the start of the LHAR structure which is expected since there is a physical step in the substrate at that area, and at the end of the conformal area where the thickness of the film is quickly reducing.

**Fig. 8:**
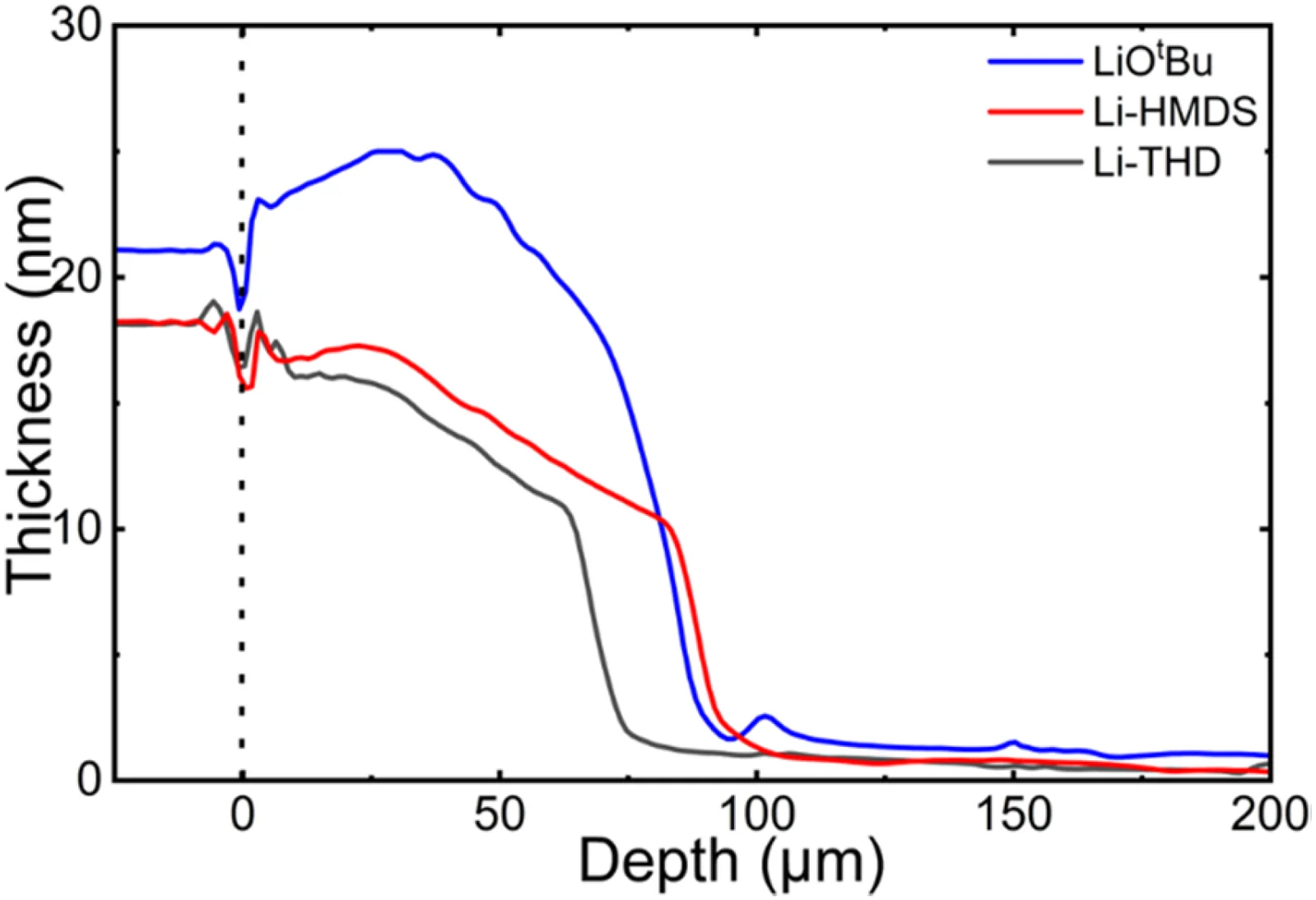
Imaging-ellipsometry-assisted thickness profiles for Li-TPA deposited on LHAR test structures with 50 ALD/MLD cycles. The dashed line at 0 distance indicating the position of the cavity entrance.

The thickness profiles for all three processes (Fig. 8) indicated somewhat similar behaviour to those previously seen for two other ALD/MLD processes: namely, for both Al-ethylene-glycol and Zn-benzene-dithiol films, an initial increase in film thickness at the membrane edge was observed, followed by a gradual decrease before an eventual drop-off.^60,61^ The difference in peak intensity observed at the zero distance resulted from the height difference between the open area and the cavity region (physical step). Most importantly, the thickness profile pattern was found to be in agreement with the SEM images as the measured PD value from SEM was apparently closer to the distance at which the thickness drop-off was observed, and the growth area appeared lighter in colour (second front).

For both the Li-HMDS + TPA and Li-THD + TPA processes, the film thickness at the membrane edge where the cavity begins is very close to the thickness in the opening area (≈18 nm) but then slightly dropped before increasing again and gradually decreasing. Though the thickness inside the cavity did not continue exactly as in the opening area, the thickness profile pattern in case of Li-HMDS showed a better conformality as the film thickness remained at 17 ± 1 nm for a distance of 37 µm inside the cavity corresponding to an aspect ratio as high as 74; this value is very promising considering the targeted 3D-microbattery application. Even though the Li-THD + TPA process displayed a very similar thickness profile pattern, the PD was found shorter and the film thickness was slightly lower than in the case of the Li-HMDS + TPA process.

The thickness profile for the LiO^*t*^Bu + TPA process was notably different from those of the other two processes, highlighting the significant influence of the precursor on the film conformality within 3D trenches and cavities. The observed film thickness at the opening area was 21 nm and increased steadily within the cavity to a maximum value of 25 nm at 26 µm, remaining then in the range of 21–25 nm up to 57 µm before decreasing abruptly. This observation of near-constant thickness plateau resembles the behaviour previously reported for an ALD/MLD process based on the relatively small trimethylaluminum (TMA) precursor; for the TMA + ethylene-glycol process, a so-called “reservoir effect” of TMA was found to cause a CVD (chemical vapor deposition) type film-growth component.^61^

For the LiO^*t*^Bu case, we carried out additional experiments using longer pulses (20 s) to investigate the PD dependency on the Li precursor pulse length. In line with previous observations for other ALD and ALD/MLD processes,^60–62^ the PD for the LiO^*t*^Bu + TPA process was slightly improved by increasing the precursor pulse length, from 84 µm (7 s) to 97 µm (20 s) (Fig. S12, SI). Moreover, the thickness profile followed a similar pattern to the 7 s case indicating the pulse length of the precursor does not significantly affect the profile shape but only controls the PD. Similar observations were made for the other two precursors. Our initial LHAR experiments using non-optimized reaction parameters (5 s Li-precursor/5 s N_2_/10 s TPA/30 s N_2_) and 100 ALD/MLD cycles at a deposition temperature of 200 °C also resulted in similar thickness profile shapes and PD trend (Fig. S12 and S13, SI) indicating the consistency in the film growth behaviour inside high-aspect-ratio structures for these lithium precursors. For the 200 °C depositions, the PD values measured from the SEM images were 23.6, 39.1, and 54.8 µm for Li-THD, LiO^*t*^Bu, and Li-HMDS, respectively. The reduction in the PD value observed might be related to the shorter pulse length and/or increased number of ALD/MLD cycles applied. The reduced pulse length could limit the precursor diffusion deep inside the cavity while the higher number of deposition cycles increases the film thickness and correspondingly lower the gap height.^62^ Most importantly the Li-HMDS based process showed longer PD values similar to the results from experiments with optimized reaction parameters. The similar trend in PD for Li-HMDS at both 200 and 220 °C deposition temperature indicates that deposition temperature didn't play a crucial role in the observed PD trend with these precursors.

**Fig. 9:**
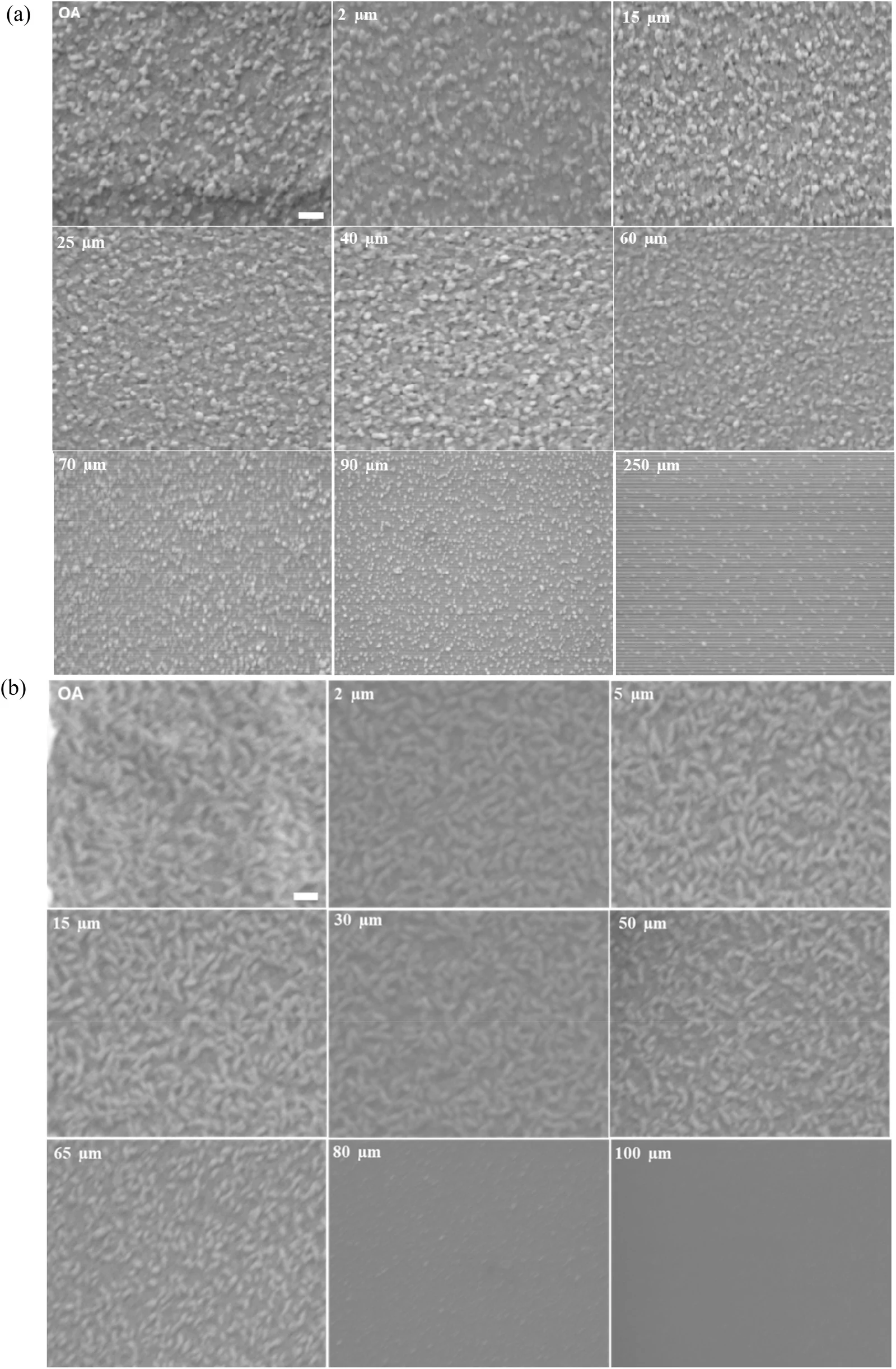
Surface morphology and structural features of Li-TPA LHAR samples deposited from (a) Li-HMDS and (b) Li-THD. The location in the cavity where the image is captured is given as a distance from the membrane edge. The image from the opening is marked with OA. The locations have an error margin of ±2 µm. The scale bar is 100 nm.

**Fig. 10:**
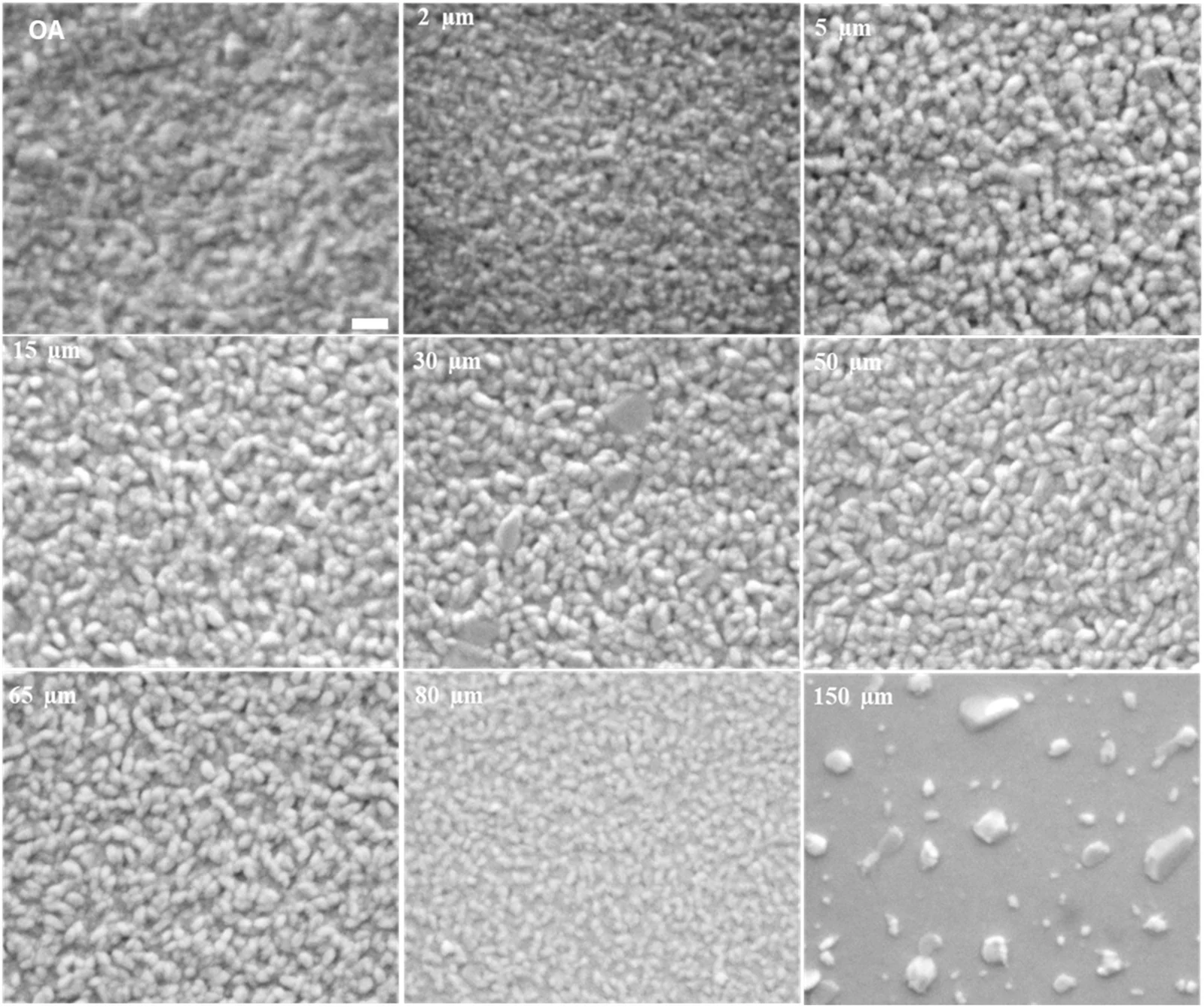
Surface morphology and structural features of Li-TPA LHAR samples deposited from LiO^*t*^Bu: the SEM images are displayed as a function of distance from the membrane edge. The image from opening is marked with OA. The locations have an error margin of ±2 µm. The scale bar is 100 nm.

To further investigate the film growth characteristics in the LHAR structures, we collected SEM images both from the opening area and inside the cavity. For the Li-HMDS case, these SEM images (Fig. 9a) revealed very similar grain aggregation patterns both in the opening area and up to *ca.* 60 µm inside the cavity, indicating the continuous nature of the film. Beyond this, the SEM images showed an increase of voids between the grains and consequently a decline in the film continuity. Grains were still visible at the film termination region (∼90 µm; Fig. 9a), but the voids were much larger, indicating that the film was no longer continuous and instead consisted of discrete grains or small islands. We examined the cavity further deep and identified very small sized grains even up to *ca.* 250 µm. For the Li-THD case (Fig. 9b), the grains appeared similar in size and morphology both in the opening area and up to *ca.* 65 µm inside the cavity. In comparison to Li-HMDS, the grains were found more distinct in appearance. The observation matches very well with the planar substrate case. The grains become smaller in size with penetration depth in such a way they were difficult to locate already at 80 µm and no grains or particles appeared at a distance of 100 µm, indicating a smooth ending of the film. The LiO^*t*^Bu based process (Fig. 10) also exhibited a similar trend in grains formation both in the opening area and inside the cavity. Similar to Li-HMDS, the grains were more crowded both in the opening area and in the beginning of the cavity but later (>60 µm) the separation between the grains started to increase. The grains also appeared smaller with more voids between them. Similar to the Li-HMDS, these grains were still visible even after the PD. However, the grains seen were bigger, and we speculate they were not Li-TPA but possibly lithium carbonates or lithium hydroxides resulting from the air exposure of unreacted LiO^*t*^Bu in the cavities. The hexameric oligomer in case of LiO^*t*^Bu could dissociate inside the cavity and since the partial pressure is reduced with decreasing in oligomeric size, these smaller fragments could travel further deep inside the cavity and get trapped there. These randomly distributed particles are one possible reason behind the observed humps in the thickness profile pattern.

To get a better understanding of the observed conformality trends, we calculated the most likely oligomeric forms of the three lithium precursors under the present ALD/MLD conditions. The possible hexameric formation in case of LiO^*t*^Bu, could agree with the observed CVD-type growth inside the cavity and a kind of reservoir effect, as the higher number of monomer units per precursor provides an excessive pool of lithium atoms for incoming TPA. On the other hand, the lower amount of monomers in the dimeric structures predicted for the other two precursors could reduce the probability of CVD-type growth and thus yield higher degree of conformality.

**Fig. 11:**
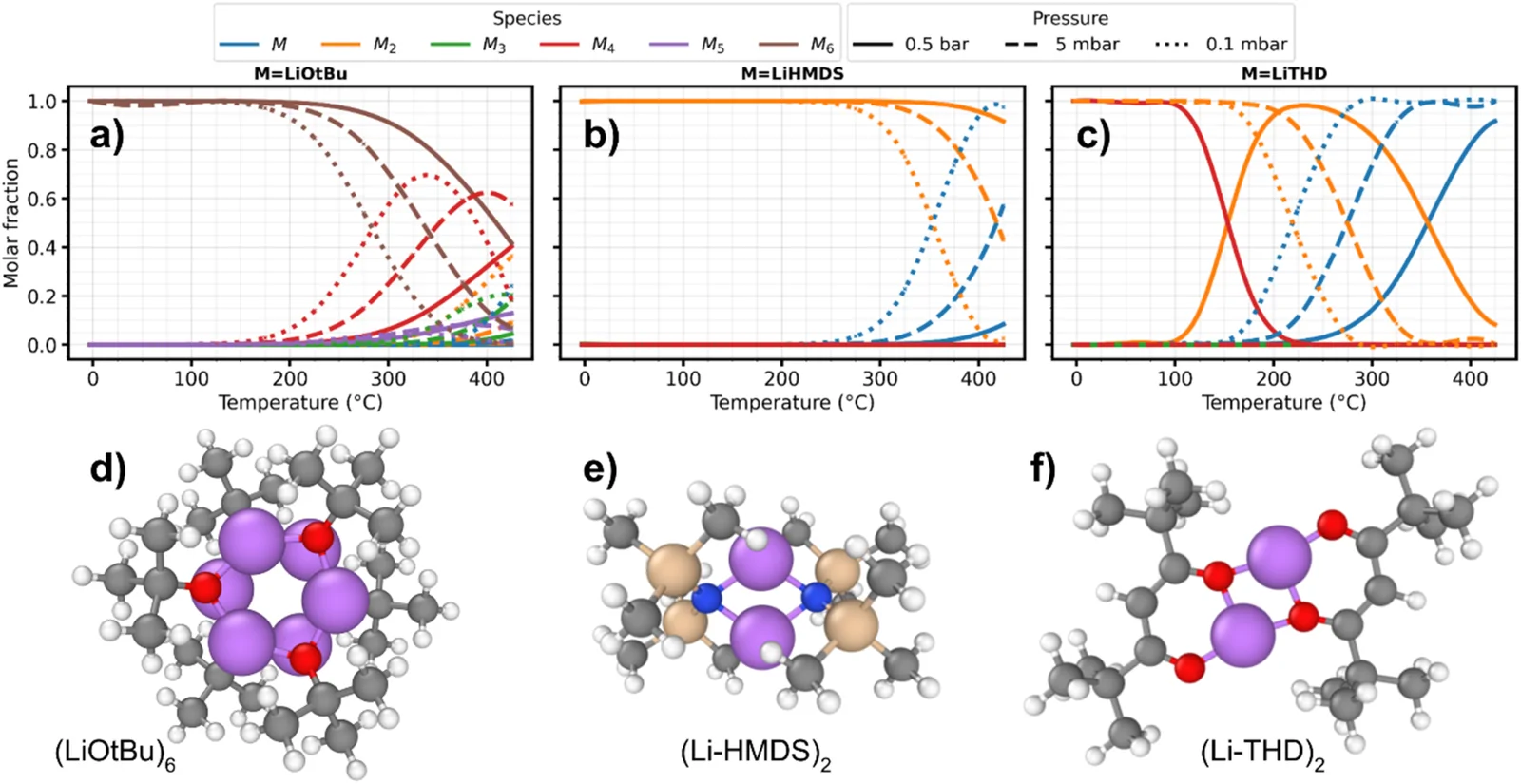
Calculated equilibrium distributions of precursor oligomers (Mn) for (a) LiO^*t*^Bu, (b) Li-HMDS, and (c) Li-THD at 0.5 bar, 5 mbar and 0.1 mbar from −3 to 425 °C. The most prevalent oligomers for each precursor at our experimental conditions are shown for LiO^*t*^Bu (d), Li-HMDS (e), and Li-THD (f).

From the calculated equilibrium distributions shown in Fig. 11, it becomes apparent that the LiO^*t*^Bu precursor is indeed present in hexameric form, (LiO^*t*^Bu)_6_, at the experimental conditions, which is in agreement with previous literature.^36^ The smaller oligomers, such as the pentamer and tetramer start becoming more prevalent at low pressures (0.1 mbar) and high temperatures (>300 °C). Note that the tetramer presence increases more than the pentamer presence with temperature, as it has a higher stabilization energy per monomer. The calculated equilibrium distributions and the oligomeric structure confirm our speculations regarding the particle formation deep inside the cavities. The ball-like structure in case of hexameric oligomer of LiO^*t*^Bu, where lithium is fully encapsulated by bulky alkyl groups might be kinetically hampering its reactivity, which allows this precursor to travel deep inside the cavity where it eventually breaks down during subsequent pulses into smaller clusters or monomers that travel deep inside the cavities and could eventually react with moisture or CO_2_ to result in carbonates or hydroxides and give rise to the particle appearance on SEM. This would also be consistent with LiO^*t*^Bu having possibly a lower sticking coefficient than the other Li precursors. For Li-HMDS, the most prevalent oligomeric form is the dimer, (Li-HMDS)_2_, which has been suggested in theoretical studies in the past.^38^ Finally, the Li-THD precursor, which is present as a dimer, is much more sensitive than LiO^*t*^Bu and Li-HMDS to changes in temperature due to a larger entropic correction to the total energy (SI), as can be seen in the lower crossing points between tetramer and dimer. From Fig. 11, it is clearly visible that compared to the (LiO^*t*^Bu)_6_ and (Li-HMDS)_2_ cases, lithium atoms in (Li-THD)_2_ are more exposed to the surroundings and we therefore surmise that they are more prone to react with the surface rather than diffusing as deep into the cavity. This reduces the possibility for crowding of this precursor inside the cavity. This is one plausible explanation for the experimentally observed appearance of grains deeper in the cavity for both LiO^*t*^Bu and Li-HMDS but not for Li-THD.

According to our calculations, summarized in Table 1, the effective diameters (*d*_eff_) of the oligomers increase in the following order: (Li-HMDS)_2_ < (LiO^*t*^Bu)_6_ < (Li-THD)_2_, corresponding to decreasing mean free paths (*λ*). The effective diameter for terephthalic acid (8.2 Å)^63^ is comparatively lower than the lithium precursor oligomers. Most importantly the *λ* for all precursors is less than the LHAR pore diameter, which indicates the gas flow in the current process is governed by viscous flow,^58^ where the particle–particle interactions are preferred over particle–surface interactions. So, a higher *λ* favours precursor diffusion deeper inside the cavity. These correlate well with the observed penetration depths, where Li-THD penetrates the least, while Li-HMDS reaches the deepest into the cavity. For a more accurate estimate of the film penetration depth, we would need to employ simulation protocols similar to what were used by Ylilammi *et al.*,^64^ but this is out of scope for this study. However, based on our results, we propose that for certain large oligomeric precursors such as those considered here, one would need to account for their shape in addition to their diameter when calculating collision cross sections, as a purely geometric measure will not be able to capture the collision dynamics of elongated rod-like structures such as (LiTHD)_2_ accurately. Nevertheless, our results suggest that it might be possible to tune the form of an ALD precursor by adjusting the reactor and sample sublimation conditions, which in turn affects the ALD chemistry.^34^ This represents a promising direction for future studies, especially in the context of area-selective ALD.

**Table 1:** Radius of gyration (*R*_g_), effective diameter (*d*_eff_) and mean free paths (*λ*) of precursor oligomers

	(LiO*t*Bu)_6_	(LiHMDS)_2_	(LiTHD)_2_
*R*_g_ (Å)	3.80	3.33	4.28
*d*_eff_ (Å)	10.01	9.06	10.97
*λ* (nm)	**208**	**284**	**130**

## Conclusions

We have demonstrated the possibility to deposit high-quality Li-TPA thin films using lithium *tert*-butoxide (LiO^*t*^Bu) and lithium bis(trimethylsilyl)amide (Li-HMDS), in addition to the previously optimized ALD/MLD process based on lithium 2,2,6,6-tetramethyl-3,5-heptanedionate (Li-THD). Through a thorough structural/chemical characterization, we were able to show the influence of the lithium precursor on the properties of the Li-TPA thin films. The precursor influence was visible in the crystallinity, morphology and surface roughness of the films.

The effect of the precursor on conformality and film penetration was investigated using lateral high-aspect-ratio test structures. These studies showed that results from planar substrates are replicable in 3D structures, which could facilitate high-surface-area 3D microbattery architectures with vastly improved rate capability and charge capacity, an important target of thin-film electrode materials such as Li-TPA. For accurately quantifying the conformality, the thickness profiles were measured using an imaging ellipsometry technique that enables film thickness determination even at Ångström level. Out of the three lithium precursors studied, Li-HMDS was found to yield the longest penetration depth and the best conformality. The experimental results were further explained through DFT optimizations combined with highly accurate DLPNO-CCSD(T) energy calculations. According to the computational results, all three precursors exist in the oligomeric form under the ALD/MLD conditions. However, both the effective diameter (*d*_eff_) and mean free path (*λ*) of oligomers displayed an inverse relation. While *d*_eff_ was (Li-HMDS)_2_ < (LiO^*t*^Bu)_6_ < (Li-THD)_2_, the *λ* was (Li-HMDS)_2_ > (LiO^*t*^Bu)_6_ > (Li-THD)_2_. Both the smaller *d*_eff_ and longer *λ* of (Li-HMDS)_2_ played a crucial role in the higher penetration depth for Li-HMDS, whereas the shorter PD with Li-THD could correlate with the higher *d*_eff_ and shorter *λ* of (Li-THD)_2_.

Additionally, SEM analysis was carried out for LHAR structures to visualize the surface morphology both in the opening area and cavity. The influence of the oligomeric structures on controlling the film's surface morphology within the cavity was evident: both LiO^*t*^Bu and Li-HMDS showed particle formation deep inside the cavity, whereas Li-THD yielded a clean film termination. Our studies indicated that Li-HMDS is the best choice among the studied lithium precursors for both planar and 3D-geometry applications. In addition to optimizing Li-TPA ALD/MLD using LiO^*t*^Bu and Li-HMDS and identifying the optimal lithium precursor in terms of conformality, our results demonstrate that computational screening can streamline precursor selection, cutting experimental work and expenses.

## Author contributions

The manuscript was written through contributions of all authors. All authors have given approval to the final version of the manuscript.

## Conflicts of interest

There are no conflicts to declare.

## Supplementary Material

NR-018-D6NR01994C-s001

## Data Availability

The computations associated with this manuscript are openly available in Zenodo, https://zenodo.org/records/19492285. Supplementary information (SI) is available. The supplementary information contains the following: Imaging Ellipsometry fitting procedure, additional refractive index data, additional FTIR, GIXRD, and SEM data, the relaxed oligomeric structures of the precursors; and additional details of the performed computations, including the procedure for calculating oligomer equlibrium distributions. See DOI: https://doi.org/10.1039/d6nr01994c.
