# Supplementary material for: Conformality of atomic/molecular layer deposited lithium terephthalate thin films
Source: Nanoscale. 2026 Sep 8;18(38):20727–39. doi: 10.1039/d6nr01994c (PMC13575822; doi:10.1039/d6nr01994c)
Supplement: Supplementary file 1 [file NR-018-D6NR01994C-s001.pdf]

Supplementary Information (SI) for Nanoscale.
This journal is © The Royal Society of Chemistry 2026

Supporting Information: Conformality of Atomic/Molecular Layer Deposited Lithium Terephthalate Thin Films

Anish Philip,[a#*] Milad Madadi,[a#] Joakim S. Jestilä,[a] Antti J. Karttunen,[a] Jussi Kinnunen,[b] and Maarit Karppinen[a*]

[a]*Department of Chemistry and Materials Science, Aalto University, FI-00076 Espoo, Finland. Emails: anish.philip@aalto.fi; maarit.karppinen@aalto.fi*

[b]*Chipmetrics Ltd, 80130, Joensuu, Finland.*

[#]*Equal contribution*

Imaging Ellipsometry fitting procedure

The optical model used is simple stack with a fixed native SiO_2 on top of single-crystal silicon substrate. The native oxide thickness is fitted using the LHAR cavity area without the Li-TPA. Subsequentially, the effective refractive index of Li-TPA is fitted at the opening region of the LHAR substrate. The crystallinity or surface roughness is not taken into account for the refractive index, instead, these are embedded to the curves as an effective refractive index. This method enables reliable film penetration depth profile evaluation. The custom Sellmeier fit made in the opening and in the cavity resulted in a low RMSE. For this reason, no additional roughness layers were added in the optical model. The imaging ellipsometer spot size behaves differently than in conventional ellipsometry. The sample is illuminated with a big, collimated light but since the sample is imaged, the resolution is determined using the camera pixels. With the used 5X objective, the "spotsize" i.e. ellipsometric resolution is 4 µm. The penetration depth profiles are extracted from the ellipsometry pictures by vertically binning multiple rows through the middle of two pillar lines.

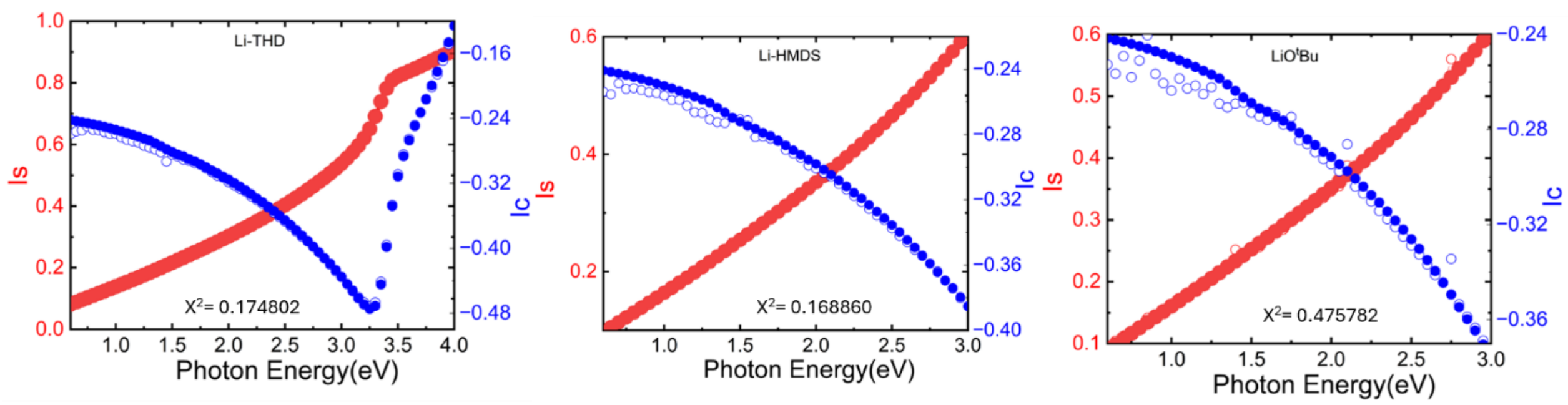

Figure S1: Ellipsometric fit data for Li-TPA deposition using three different lithium precursors. Hollow circles correspond to the measured data, while solid circles correspond to the fit. Measured data corresponds to the variables Is = sin(2Ψ) sin(Δ) and Ic = sin(2Ψ) cos(Δ), respectively shown in red and blue. χ^2 value is specified in the graph.

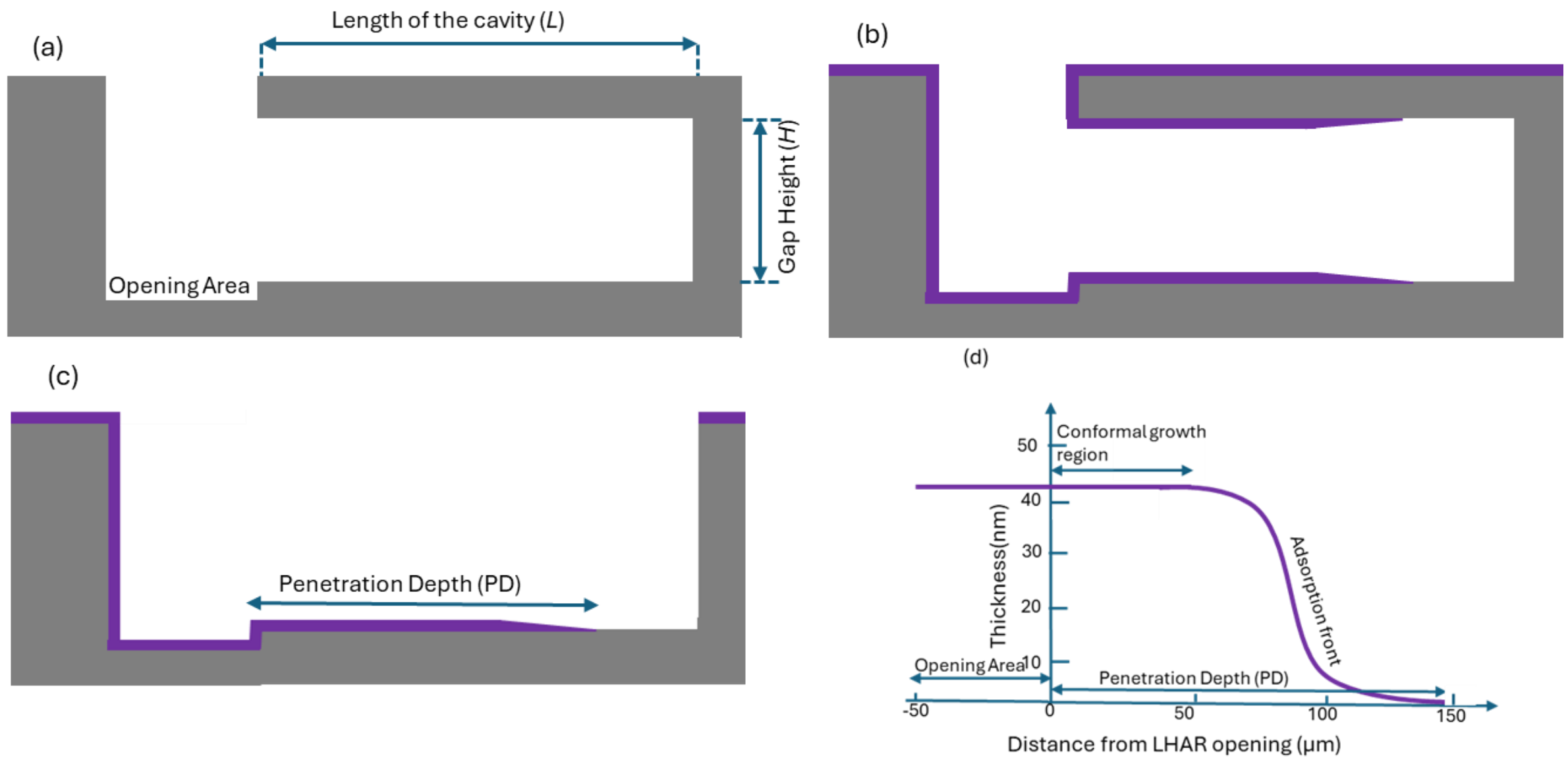

Figure S2. Schematic illustration of the conformality analysis using PillarHall LHAR test structures. Test structure (a) before ALD coating, (b) after ALD coating, and (c) after ALD coating and peeling off the roof membrane, and (d) thickness profile explanation. L and H stand for cavity length and gap height, respectively (nominal gap height: 500 nm). Both opening area and penetration depth (PD) are marked in the schematics. The adsorption front of thickness profile is also indicated.

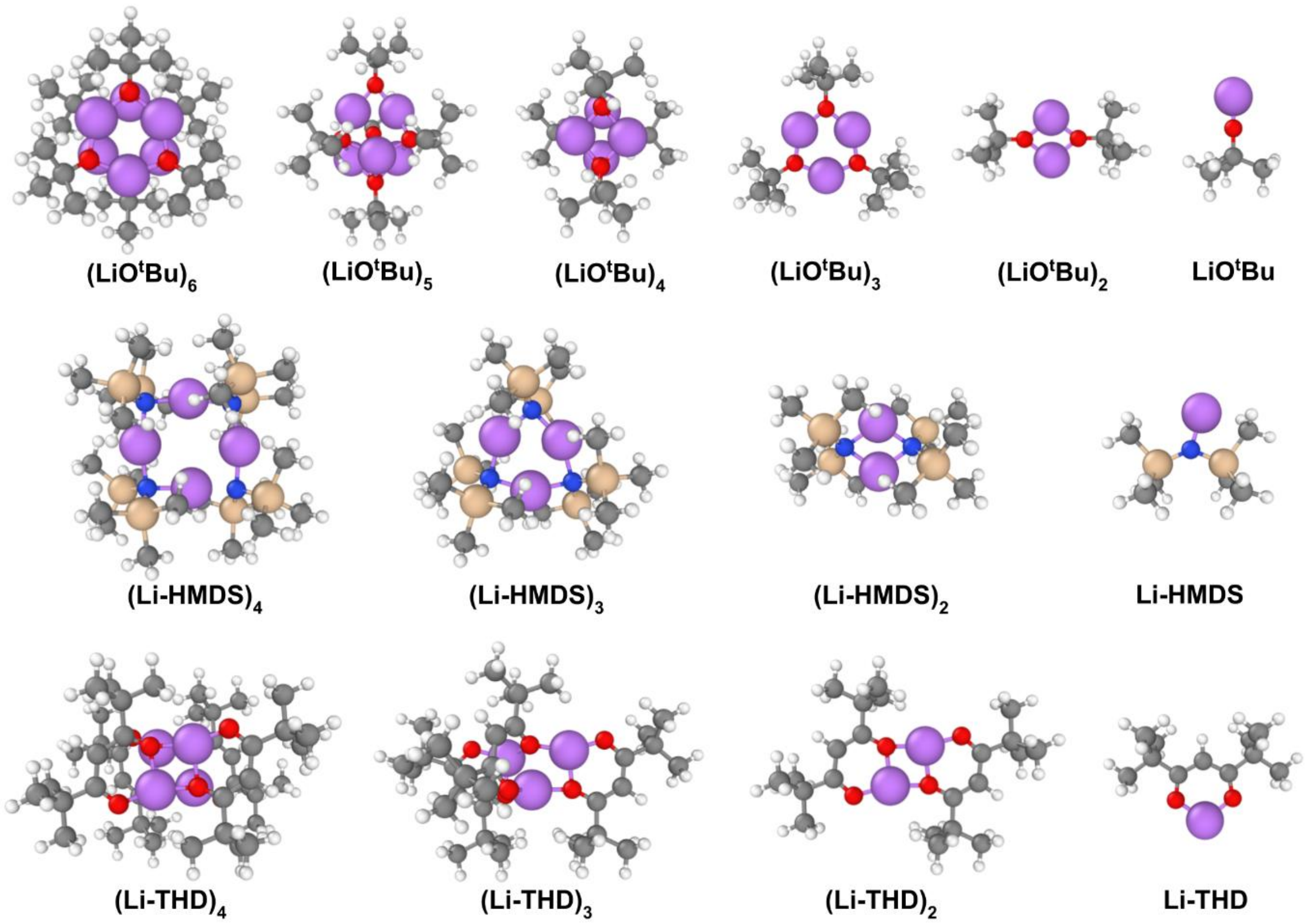

Figure S3. Relaxed oligomeric structures obtained using an initial conformer search with CREST followed by re-optimization of the lowest energy structures with B3LYP-D4/Def2-TZVPP

Details of computational calculations performed

B3LYP-D4/Def2-TZVPP oligomer energies and thermal corrections (at 298.15 K and 1 atm)

Table S1. LiO^{t}Bu oligomer (M_n) B3LYP-D4/Def2-TZVPP optimized energies and thermal corrections (Hartrees)

Oligomer (n)	Electronic energy (E_{el})	ZPE	Gibbs free energy (G)	Enthalpy	Entropy correction (multiplied by T = 298.15 K)	G-E_{el}
**1**	-240.594694	0.12506839	-240.500119	-240.461032	0.03908675	0.09457523
**2**	-481.282383	0.25213913	-481.06913	-481.014712	0.05441843	0.21325331
**3**	-721.949962	0.37819696	-721.624082	-721.545822	0.07826022	0.32588054
**4**	-962.642037	0.50676916	-962.190716	-962.102895	0.08782106	0.45132143
**5**	-1203.31248	0.6344925	-1202.74307	-1202.63517	0.10790372	0.56940413
**6**	-1444.00256	0.76066161	-1443.31249	-1443.19203	0.12046073	0.69006904

Table S2. Li-HMDS oligomer (M_n) B3LYP-D4/Def2-TZVPP optimized energies and thermal corrections (Hartrees)

Oligomer (n)	Electronic energy (E_{el})	ZPE	Gibbs free energy (G)	Enthalpy	Entropy correction (multiplied by T = 298.15 K)	G-E_{el}
**1**	-880.801713	0.22729974	-880.616799	-880.555806	0.06099246	0.18491423
**2**	-1761.68802	0.45821837	-1761.29015	-1761.19293	0.0972203	0.39787705
**3**	-2642.54725	0.69056041	-2641.93259	-2641.80178	0.13080908	0.61465485
**4**	-3523.39884	0.92201587	-3522.56899	-3522.40344	0.16554927	0.8298492

Table S3. Li-THD oligomer (M_n) B3LYP-D4/Def2-TZVPP optimized energies and thermal corrections (Hartrees)

Oligomer (n)	Electronic energy (E_{el})	ZPE	Gibbs free energy (G)	Enthalpy	Entropy correction (multiplied by T = 298.15 K)	G-E_{el}
**1**	-588.6011465	0.28204224	-588.3603652	-588.3016183	0.05874691	0.24078136
**2**	-1177.263219	0.5657367	-1176.75787	-1176.661636	0.09623429	0.50534926
**3**	-1765.907767	0.85079796	-1765.132477	-1765.003724	0.12875298	0.77528969
**4**	-2354.580709	1.13492235	-2353.535661	-2353.374642	0.16101886	1.04504877

DLPNO-CCSD(T)/CBS oligomer energies with thermal corrections (at 298.15 K and 1 atm)

Table S4. LiO^{t}Bu oligomer (M_n) DLPNO-CCSD(T)/CBS (3/4, Def2-TZVPP/Def2-QZVPP extrapolation) energies with B3LYP-D4/Def2-TZVPP thermal corrections (Hartrees)

Oligomer (n)	Electronic energy (E_{el})	Gibbs free energy with B3LYP corrections (G)	G-E_{el}
**1**	-240.3410573	-240.246482	0.09457523
**2**	-480.7753575	-480.5621042	0.21325331
**3**	-721.1902274	-720.8643469	0.32588054
**4**	-961.6354706	-961.1841491	0.45132143
**5**	-1202.054048	-1201.484644	0.56940413

**6**	-1442.49008	-1441.800011	0.69006904

Table S5. Li-HMDS oligomer (M_n) DLPNO-CCSD(T)/CBS (3/4, Def2-TZVPP/Def2-QZVPP extrapolation) energies with B3LYP-D4/Def2-TZVPP thermal corrections (Hartrees)

**Oligomer (*n*)**	**Electronic energy (E_{el})**	**Gibbs free energy with B3LYP corrections (*G*)**	***G*-E_{el}**
**1**	-879.6822879	-879.4973737	0.18491423
**2**	-1759.450249	-1759.052372	0.39787705
**3**	-2639.188663	-2638.574008	0.61465485
**4**	-3518.919131	-3518.089282	0.8298492

Table S6. Li-THD oligomer (M_n) DLPNO-CCSD(T)/CBS (3/4, Def2-TZVPP/Def2-QZVPP extrapolation) energies with B3LYP-D4/Def2-TZVPP thermal corrections (Hartrees)

**Oligomer (*n*)**	**Electronic energy (E_{el})**	**Gibbs free energy with B3LYP corrections (*G*)**	***G*-E_{el}**
**1**	-587.9523047	-587.7115234	0.24078136
**2**	-1175.966377	-1175.461027	0.50534926
**3**	-1763.964151	-1763.188861	0.77528969
**4**	-2351.989362	-2350.944313	1.04504877

Thermal corrections at a range of temperatures (-3 to 425 °C) from B3LYP-D4/Def2-TZVPP

Table S7. Thermal corrections for the LiO^{t}Bu oligomers (M_n)

**Temperature in °C**	**Temperature (K)**	***n*=1**	***n*=2**	***n*=3**	***n*=4**	***n*=5**	***n*=6**
-3.15	270	0.09818007	0.21811265	0.33268366	0.45891433	0.5785792	0.70025225
25	298.15	0.09457524	0.2132533	0.32588054	0.45132143	0.56940412	0.69006904
75	348.15	0.08787367	0.20406397	0.31293326	0.43667599	0.55162985	0.67023449
125	398.15	0.08080188	0.19417441	0.29890671	0.4205788	0.53200897	0.64821244
175	448.15	0.07337181	0.1836007	0.28382919	0.4030664	0.51059149	0.62405892
225	498.15	0.06559445	0.17235959	0.26772889	0.38417641	0.48742766	0.59783137
275	548.15	0.05748045	0.16046864	0.25063451	0.36394767	0.46256853	0.56958888
325	598.15	0.04904023	0.14794595	0.23257512	0.34241964	0.43606543	0.53939132
375	648.15	0.04028399	0.1348098	0.21357965	0.3196317	0.40796923	0.50729832
425	698.15	0.03122164	0.12107828	0.19367655	0.29562248	0.3783296	0.47336832

Table S8. Thermal corrections for the Li-HMDS oligomers (M_n)

**Temperature in °C**	**Temperature (K)**	***n*=1**	***n*=2**	***n*=3**	***n*=4**
-3.15	270	0.19036516	0.40630941	0.62587116	0.84393142
25	298.15	0.18491423	0.39787703	0.61465486	0.8298492
75	348.15	0.1746084	0.38165703	0.59286154	0.80233682
125	398.15	0.16353622	0.36390686	0.56876326	0.77174523
175	448.15	0.15173178	0.34469183	0.54245792	0.7382055
225	498.15	0.13922655	0.32407336	0.51403787	0.70184132
275	548.15	0.12604988	0.30210937	0.4835904	0.66276957
325	598.15	0.11222919	0.27885426	0.45119758	0.62110016
375	648.15	0.09778999	0.25435889	0.41693621	0.57693588

425	698.15	0.08275599	0.22867061	0.3808778	0.53037246

Table S9. Thermal corrections for the Li-THD oligomers (M_n)

Temperature in °C	Temperature (K)	*n*=1	*n*=2	*n*=3	*n*=4
-3.15	270	0.24603021	0.5136513	0.78622225	1.05860675
25	298.15	0.24078136	0.50534925	0.77528969	1.04504877
75	348.15	0.23082863	0.48935095	0.75399298	1.01844736
125	398.15	0.2200858	0.47177972	0.73033436	0.98867987
175	448.15	0.20857021	0.45266882	0.70436229	0.95581351
225	498.15	0.19629973	0.43205305	0.67612834	0.91991913
275	548.15	0.18329324	0.40996952	0.64568799	0.88107232
325	598.15	0.16957042	0.38645711	0.61309961	0.83935221
375	648.15	0.15515138	0.36155564	0.57842305	0.79483966
425	698.15	0.14005622	0.33530499	0.54171833	0.74761556

Equilibrium constant and partial pressure calculation details

The Gibbs free energies for the fragmentation of oligomers M_n to their constituent monomers M,

$$M_n\ (g) \rightarrow n\ M\ (g)$$

where n indicates the degree of oligomerization (e.g. $n = 2$, dimerization), were computed as follows:

$$\Delta_{frag}G° = n\Delta G°_M - \Delta G°_{M_n}$$

where $\Delta G°_M$ is the Gibbs free energy of the monomer, while $\Delta G°_{M_n}$ is the Gibbs free energy for the *n*-oligomer. From these, equilibrium constants at a range of temperatures from -3 to 425 °C for the monomer-oligomer equilibrium were determined according to:

$$\Delta_{frag}G° = -RTlnK_n$$

$$K_n = e^{\frac{-\Delta_{frag}G°}{RT}}$$

where K_n is the equilibrium constant for the dissociation of an *n*-oligomer into monomers, where we assume that each oligomer is in equilibrium through their monomers. Then, finally, we can solve the molar fractions of each oligomer at a given pressure and temperature by noting that the equilibrium constant doesn't change with pressure (while the partial pressures of gas mixture components do):

$$K_n = \frac{P_1^n}{P_n}$$

$$P_n = \frac{P_1^n}{K_n}$$

and setting up the total pressure as a function of the partial pressures of each oligomer:

$$P_{total} = P_1 + P_2 + P_3 + \cdots + P_n$$

which can be substituted by the expression for P_n to give an equation for the partial pressures that can be solved according to the monomer pressure:

$$P_{tot} = P_1 + \frac{P_1^2}{K_2} + \frac{P_1^3}{K_3} + \cdots + \frac{P_1^n}{K_n}$$

In the case there is a carrier gas, the equation becomes:

$$P_{tot} = P_1 + \frac{P_1^2}{K_2} + \frac{P_1^3}{K_3} + \cdots + \frac{P_1^n}{K_n} + P_{N_2}$$

where P_{N_2} can be substituted by a fraction of P_{tot}.

Gibbs free energies of fragmentation into monomers ($\Delta_{frag}G°$) computed with DLPNO-CCSD(T)/CBS

Table S10. LiO^{t}Bu oligomer (M_n) fragmentation energies ($\Delta_{frag}G°$) computed with DLPNO-CCSD(T)/CBS energies and B3LYP-D4/Def2-TZVPP thermal corrections

Number of monomers	Total free energy of monomers (Hartrees)	Total free energy of *n*-oligomer (Hartrees)	$\Delta_{frag}G°$ (Hartrees)	$\Delta_{frag}G°$ (kJ/mol)	$\Delta\Delta_{frag}G°$ (kJ/mol)
1	-240.246482	-240.246482	n.a	n.a	n.a
2	-480.492964	-480.5621042	0.069140148	181.5274583	181.5274583
3	-720.739446	-720.8643469	0.124900837	327.9271475	146.3996891
4	-960.985928	-961.1841491	0.198221029	520.4293115	192.5021641
5	-1201.23241	-1201.484644	0.252234088	662.2405992	141.8112877
6	-1441.47889	-1441.800011	0.321118601	843.0968868	180.8562875

Table S11. Li-HMDS oligomer (M_n) fragmentation energies ($\Delta_{frag}G°$) computed with DLPNO-CCSD(T)/CBS energies and B3LYP-D4/Def2-TZVPP thermal corrections

Number of monomers	Total free energy of monomers (Hartrees)	Total free energy of *n*-oligomer (Hartrees)	$\Delta_{frag}G°$ (Hartrees)	$\Delta_{frag}G°$ (kJ/mol)	$\Delta\Delta_{frag}G°$ (kJ/mol)
1	-879.4973737	-879.4973737	*n.a*	*n.a*	*n.a*
2	-1758.994747	-1759.052372	0.057624907	151.2941928	151.2941928
3	-2638.492121	-2638.574008	0.081887013	214.9943529	63.70016012
4	-3517.989495	-3518.089282	0.099787247	261.9914176	46.99706461

Table S12. Li-THD oligomer (M_n) fragmentation energies ($\Delta_{frag}G°$) computed with DLPNO-CCSD(T)/CBS energies and B3LYP-D4/Def2-TZVPP thermal corrections

Number of monomers	Total free energy of monomers (Hartrees)	Total free energy of *n*-oligomer (Hartrees)	$\Delta_{frag}G°$ (Hartrees)	$\Delta_{frag}G°$ (kJ/mol)	$\Delta\Delta_{frag}G°$ (kJ/mol)
1	-587.711523	-587.711523	*n.a*	*n.a*	*n.a*
2	-1175.42305	-1175.46103	0.037980771	99.71851412	99.71851412
3	-1763.13457	-1763.18886	0.054291137	142.541379	42.82286488
4	-2350.84609	-2350.94431	0.098219492	257.8752761	115.3338971

Calculated equilibrium constants for oligomer (M_n) dissociation into monomers at range of temperatures (-3 to 425 °C, 1 atm)

Table S13. Equilibrium constants (K_n) for LiO^{t}Bu oligomer dissociation reactions at 1 atm

Temperature (°C)	M_2	M_3	M_4	M_5	M_6
-3.15	4.87859E-37	3.32992E-66	7.1047E-105	2.4529E-133	1.2097E-169
25	1.57671E-32	3.54516E-58	6.67899E-92	9.5596E-117	1.976E-148
75	2.66029E-26	4.16694E-47	5.1195E-74	6.92358E-94	3.4226E-119
125	1.26453E-21	8.32541E-39	1.29382E-60	9.12518E-77	2.76037E-97
175	5.56187E-18	2.37744E-32	3.38329E-50	1.81314E-63	2.91688E-80
225	4.6199E-15	3.47642E-27	7.22369E-42	7.66268E-53	1.18606E-66
275	1.14108E-12	5.85941E-23	4.68363E-35	3.70503E-44	1.59138E-55
325	1.1343E-10	1.95467E-19	2.21244E-29	6.28598E-37	2.94856E-46
375	5.59632E-09	1.87605E-16	1.39503E-24	8.1243E-31	2.03095E-38
425	1.59171E-07	6.76654E-14	1.80895E-20	1.3909E-25	1.05445E-31

Table S14. Equilibrium constants (K_n) for Li-HMDS oligomer dissociation reactions at 1 atm

Temperature (°C)	M_2	M_3	M_4
-3.15	2.99663E-31	6.29093E-45	2.47612E-55
25	3.12173E-27	2.16084E-38	1.26209E-46
75	1.07385E-21	2.17809E-29	1.24102E-34
125	1.5059E-17	1.19721E-22	1.17384E-25
175	2.50737E-14	2.04709E-17	1.08826E-18
225	9.40699E-12	3.09191E-13	3.97553E-13
275	1.19509E-09	8.01013E-10	1.3861E-08
325	6.74375E-08	5.53789E-07	8.31451E-05
375	2.03932E-06	0.00013873	0.128995984
425	3.77875E-05	0.015664412	69.24222458

Table S15. Equilibrium constants (K_n) for Li-THD oligomer dissociation reactions at 1 atm

Temperature (°C)	M_2	M_3	M_4
-3.15	3.92142E-21	9.53042E-31	2.15954E-54
25	3.38897E-18	1.06618E-25	6.64058E-46
75	3.78529E-14	9.69802E-19	3.26449E-34
125	4.09189E-11	1.58719E-13	1.8492E-25
175	9.34486E-09	1.78902E-09	1.16032E-18
225	7.19601E-07	3.10429E-06	3.12807E-13
275	2.51487E-05	0.001382792	8.57319E-09
325	0.000486153	0.22230073	4.23884E-05
375	0.005960671	16.3246803	0.056209083
425	0.051109671	647.9235136	26.52864586

Calculated oligomer partial pressures (P_n) at total precursor pressure of 5 mbar for a range of temperatures (-3 to 425 °C)

Table S16. Partial pressures of the LiO^{t}Bu oligomers according to a total precursor pressure of 5 mbar (0.005 bar). Partial pressures shown in bar (P1 = monomer, P2 = dimer, P3 = trimer, P4 = tetramer, P5 = pentamer, P6 = hexamer)

Temperature (°C)	P1	P2	P3	P4	P5	P6
-3.15	2.91E-29	1.73E-21	7.39E-21	1.01E-10	8.48E-11	5.00E-03
25	9.98E-26	6.32E-19	2.80E-18	1.49E-09	1.04E-09	5.00E-03
75	7.45E-21	2.09E-15	9.93E-15	6.02E-08	3.32E-08	5.00E-03
125	3.34E-17	8.80E-13	4.46E-12	9.58E-07	4.53E-07	5.00E-03
175	2.29E-14	9.46E-11	5.07E-10	8.18E-06	3.50E-06	4.99E-03
225	4.25E-12	3.90E-09	2.20E-08	4.50E-05	1.80E-05	4.94E-03
275	3.02E-10	7.99E-08	4.69E-07	1.77E-04	6.77E-05	4.75E-03
325	1.03927E-08	9.52E-07	5.74E-06	5.27E-04	1.93E-04	4.27E-03
375	2.0197E-07	7.29E-06	4.39E-05	1.19E-03	4.14E-04	3.34E-03
425	2.46E-06	3.79E-05	2.19E-04	2.01E-03	6.43E-04	2.08E-03

Table S17. Partial pressures of the Li-HMDS oligomers according to a total precursor pressure of 5 mbar (0.005 bar). Partial pressures shown in bar (P1 = monomer, P2 = dimer, P3 = trimer, P4 = tetramer)

Temperature (°C)	P1	P2	P3	P4
-3.15	3.87E-17	4.99E-03	9.19E-06	9.03E-12
25	3.95E-15	5.00E-03	2.85E-06	1.93E-12
75	2.32E-12	5.00E-03	5.71E-07	2.32E-13
125	2.74E-10	5.00E-03	1.73E-07	4.83E-14
175	1.12E-08	5.00E-03	6.86E-08	1.44E-14
225	2.17E-07	5.00E-03	3.30E-08	5.56E-15
275	2.44E-06	5.00E-03	1.82E-08	2.57E-15
325	1.8329E-05	4.98E-03	1.11E-08	1.36E-15
375	9.99637E-05	4.90E-03	7.20E-09	7.74E-16
425	4.16E-04	4.58E-03	4.60E-09	4.33E-16

Table S18. Partial pressures of the Li-THD oligomers according to a total precursor pressure of 5 mbar (0.005 bar). Partial pressures shown in bar (P1 = monomer, P2 = dimer, P3 = trimer, P4 = tetramer)

Temperature (°C)	P1	P2	P3	P4
-3.15	1.02E-14	2.65E-08	1.11E-12	5.00E-03
25	1.35E-12	5.38E-07	2.31E-11	5.00E-03
75	1.13E-09	3.36E-05	1.48E-09	4.97E-03
125	1.68E-07	6.90E-04	2.99E-08	4.31E-03
175	6.01E-06	3.87E-03	1.21E-07	1.13E-03
225	5.94E-05	4.90E-03	6.75E-08	3.98E-05
275	3.42E-04	4.66E-03	2.90E-08	1.60E-06
325	0.001334837	3.67E-03	1.07E-08	7.49E-08
375	0.00323945	1.76E-03	2.08E-09	1.96E-09
425	4.59E-03	4.12E-04	1.49E-10	1.67E-11

Calculated oligomer partial pressures (P_n) at total reactor pressure (including 99% N_2 carrier gas) of 5 mbar for a range of temperatures (-3 to 425 °C)

Table S19. Partial pressures of the LiO^{t}Bu oligomers according to a total pressure of 5 mbar (0.005 bar). Partial pressures shown in bar (P1 = monomer, P2 = dimer, P3 = trimer, P4 = tetramer, P5 = pentamer, P6 = hexamer)

Temperature (°C)	P1	P2	P3	P4	P5	P6
-3.15	1.35E-29	3.74E-22	7.39E-22	4.68E-12	1.83E-12	5.00E-05
25	4.62E-26	1.35E-19	2.78E-19	6.82E-11	2.20E-11	4.92E-05
75	3.45E-21	4.47E-16	9.85E-16	2.77E-09	7.06E-10	4.93E-05
125	1.55E-17	1.90E-13	4.46E-13	4.45E-08	9.77E-09	5.00E-05
175	1.06E-14	2.03E-11	5.06E-11	3.78E-07	7.50E-08	4.95E-05
225	1.96E-12	8.30E-10	2.16E-09	2.04E-06	3.76E-07	4.76E-05
275	1.37E-10	1.64E-08	4.37E-08	7.48E-06	1.29E-06	4.12E-05
325	4.49511E-09	1.78E-07	4.65E-07	1.85E-05	2.92E-06	2.80E-05
375	7.99094E-08	1.14E-06	2.72E-06	2.92E-05	4.01E-06	1.28E-05
425	8.50E-07	4.53E-06	9.06E-06	2.88E-05	3.18E-06	3.57E-06

Table S20. Partial pressures of the Li-HMDS oligomers according to a total pressure of 5 mbar (0.005 bar). Partial pressures shown in bar (P1 = monomer, P2 = dimer, P3 = trimer, P4 = tetramer)

Temperature (°C)	P1	P2	P3	P4
-3.15	3.87E-18	5.00E-05	3.91E-43	1.34E-61
25	3.95E-16	5.00E-05	4.56E-39	3.61E-56
75	2.32E-13	5.00E-05	1.89E-33	1.14E-48
125	2.74E-11	5.00E-05	3.10E-29	4.90E-43
175	1.12E-09	5.00E-05	5.87E-26	1.17E-38
225	2.17E-08	5.00E-05	2.43E-23	3.70E-35
275	2.44E-07	4.98E-05	3.20E-21	2.53E-32
325	1.80286E-06	4.82E-05	1.64E-19	4.91E-30
375	9.12952E-06	4.09E-05	3.68E-18	3.16E-28
425	2.85E-05	2.15E-05	3.92E-17	7.52E-27

Table S21. Partial pressures of the Li-THD oligomers according to a total precursor pressure of 5 mbar (0.005 bar). Partial pressures shown in bar (P1 = monomer, P2 = dimer, P3 = trimer, P4 = tetramer)

Temperature (°C)	P1	P2	P3	P4
-3.15	4.43E-13	5.00E-05	2.58E-57	1.54E-62
25	1.30E-11	5.00E-05	9.25E-52	6.86E-57
75	1.38E-09	5.00E-05	4.23E-44	4.34E-49
125	4.52E-08	5.00E-05	2.34E-38	3.11E-43
175	6.79E-07	4.93E-05	6.72E-34	1.10E-38
225	5.65E-06	4.44E-05	2.42E-30	4.70E-35
275	2.50E-05	2.50E-05	1.85E-27	4.08E-32
325	4.57034E-05	4.30E-06	4.09E-25	9.63E-30
375	4.95875E-05	4.13E-07	3.13E-23	7.25E-28
425	5.00E-05	4.88E-08	9.47E-22	1.96E-26

Parameters and effective molecular size and mean free paths of most prevalent oligomers in the experiments (λ)

Table S22. Parameters used to calculate the effective molecular sizes and mean free paths of precursor oligomers

	$(LiO^tBu)_6$	$(Li\text{-}HMDS)_2$	$(Li\text{-}THD)_2$	N_2 (carrier gas)
**Mass (g/mol)**	480.32	334.65	380.42	28.014
**End-to-end distance (Å)**	10.99	9.77	13.83	1.098
**A (MHZ)**	114.53	330.18	240.76	-
**B (MHZ)**	114.20	182.88	99.001	59958.5
**C (MHZ)**	99.90	161.17	74.78	-
**I_x (amu Å^2)**	4412.63	1530.62	2099.12	-
**I_y (amu Å^2)**	4425.58	2763.44	5104.44	8.43
**I_z (amu Å^2)**	5058.99	3135.63	6758.13	-
**σ_{ii} (Å^2)**	314.60	258.07	377.89	-
**σ_{ij} (Å^2)**	145.87	126.37	260.43	-
**R_g (Å)**	3.80	3.33	4.28	0.26
**d_{eff} (Å)**	10.01	9.06	10.97	3.62
**λ (nm)**	208	284	130	-

The conversion between rotational constants (A, B, C) and moments of inertia (I_A, I_B, I_C) are given by:

$$I_A = \frac{h}{8\pi^2 A}; I_B = \frac{h}{8\pi^2 B}; I_C = \frac{h}{8\pi^2 C}$$

where *h* is the Planck constant, and rotational constants are in MHz, which gives the conversion factor 505 379.005 between [A(MHz)] and [I_a(amu-Å^2)]. These are then used to estimate the radius of gyration for the oligomers:

$$R_g = \sqrt{\frac{I_A + I_B + I_C}{2m}}$$

where *m* is the mass of the oligomer (in *amu*). On top of this, we add a small correction for the van der Waals radius of the outermost atoms (H = 1.2 Å, N = 1.5 Å), so that the effective diameter in the case of the precursors (d_{eff}) becomes:

$$d_{eff} = 2(R_g + 1.2\ \text{Å})$$

The effective diameter is used in the calculation of the collision cross section (σ) for a particle with itself,

$$\sigma_{ii} = \pi\left(d_{eff}\right)^2$$

and with the inert carrier gas (N_2):

$$\sigma_{ij} = \pi\left(\frac{d_{eff,i} + d_{eff,j}}{2}\right)^2$$

Finally, the mean free paths are calculated according to references [1,2]

$$\lambda = \frac{k_B T}{\sqrt{2} p_i \sigma_{ii} + \sqrt{1 + \frac{m_i}{m_j}} p_j \sigma_{ij}}$$

where p_i, p_j are the partial pressures (in Pa) of the precursors and carrier gases; σ_{ii}, σ_{ij} the collision cross sections (in m^2) between a molecule and itself, and between a molecule and the carrier gas; and m_i, m_j the masses (in kg) of the precursors and the carrier gas, respectively.

Additional Experimental Data

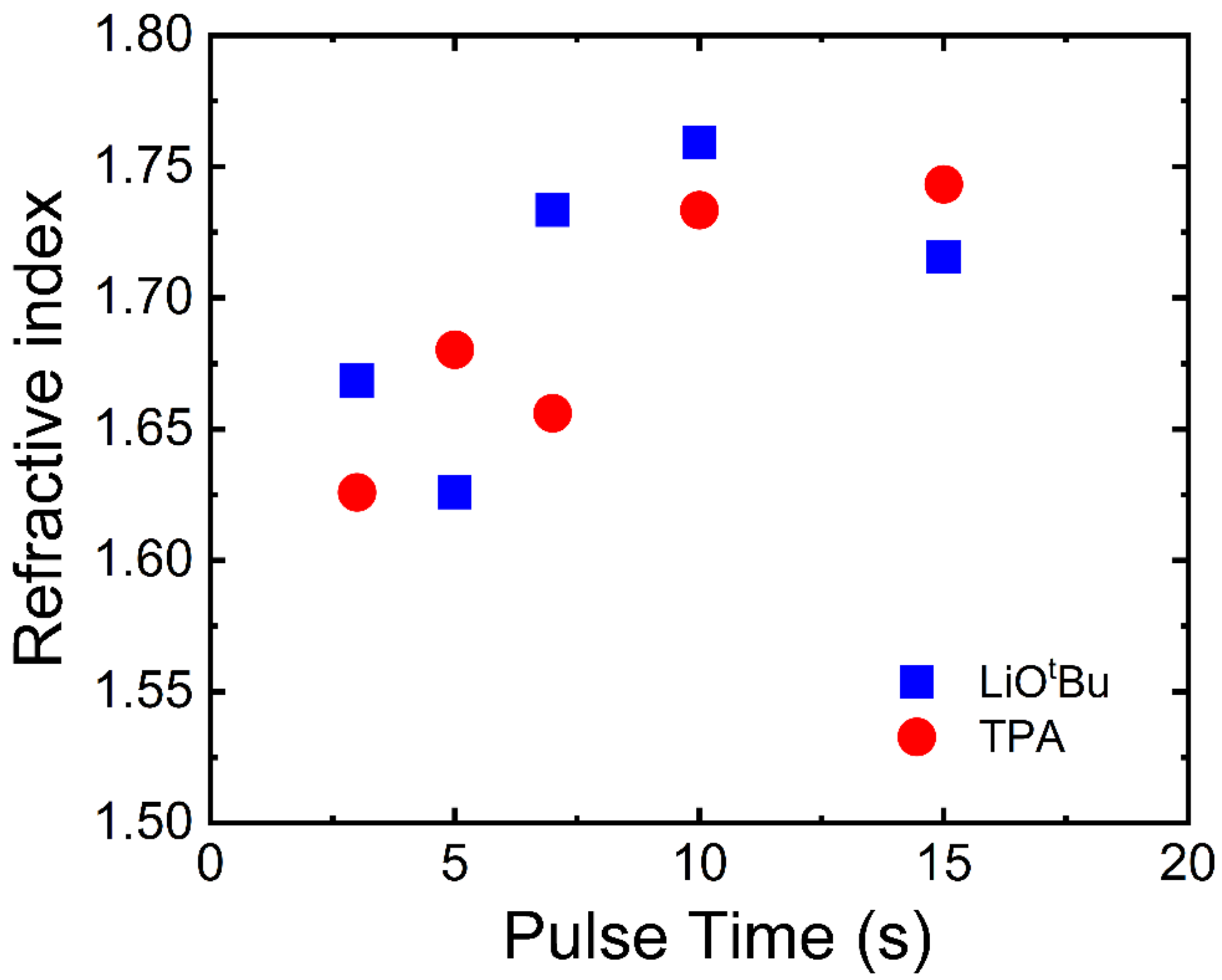

Figure S4. The change in refractive index at **λ**=633 nm observed with precursor pulse length in case of LiO^{t}Bu+TPA deposition. The refractive index values were obtained from the ellipsometry measurements.

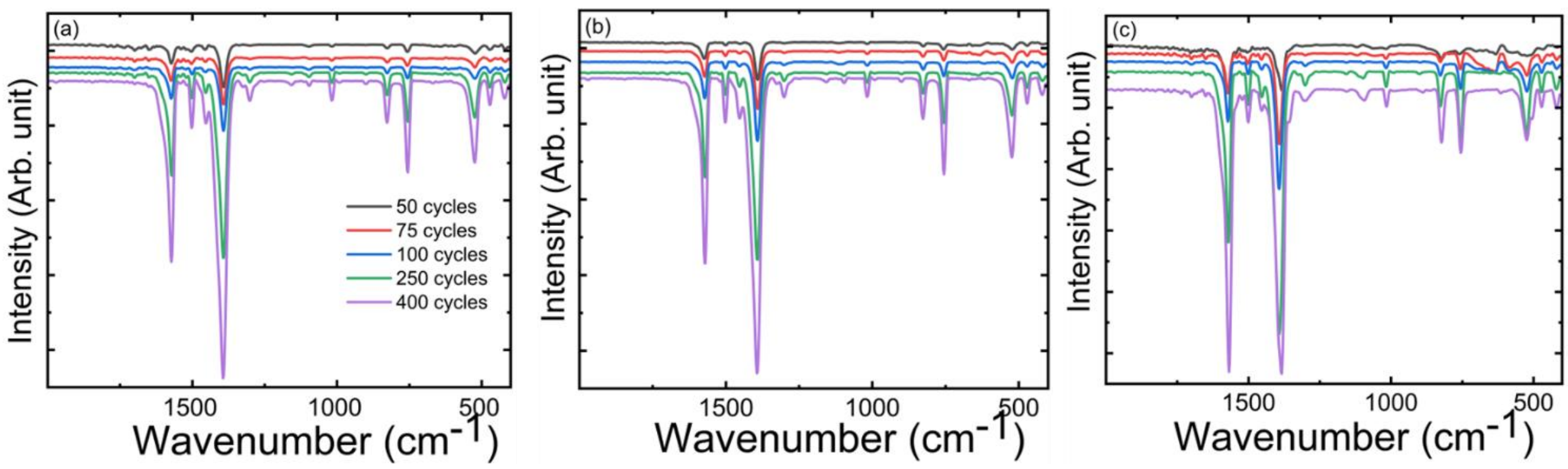

Figure S5. FTIR spectra showing the linear dependency of IR band intensity with the number of ALD/MLD cycles, (a) for Li-HMDS, (b) Li-THD and (c) LiO^{t}Bu-based Li-TPA processes.

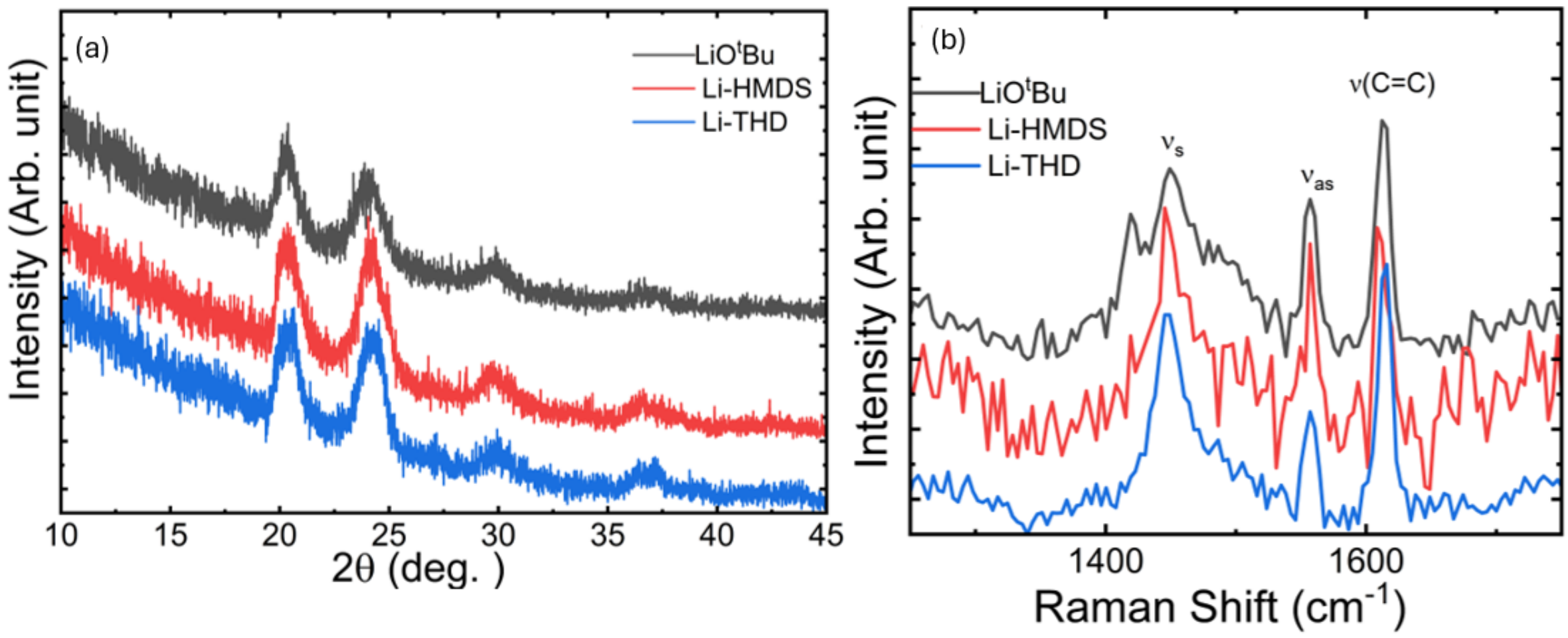

Figure S6. GIXRD (a) and Raman spectra (b) collected from Li-TPA thin films deposited with 100 cycles of ALD/MLD.

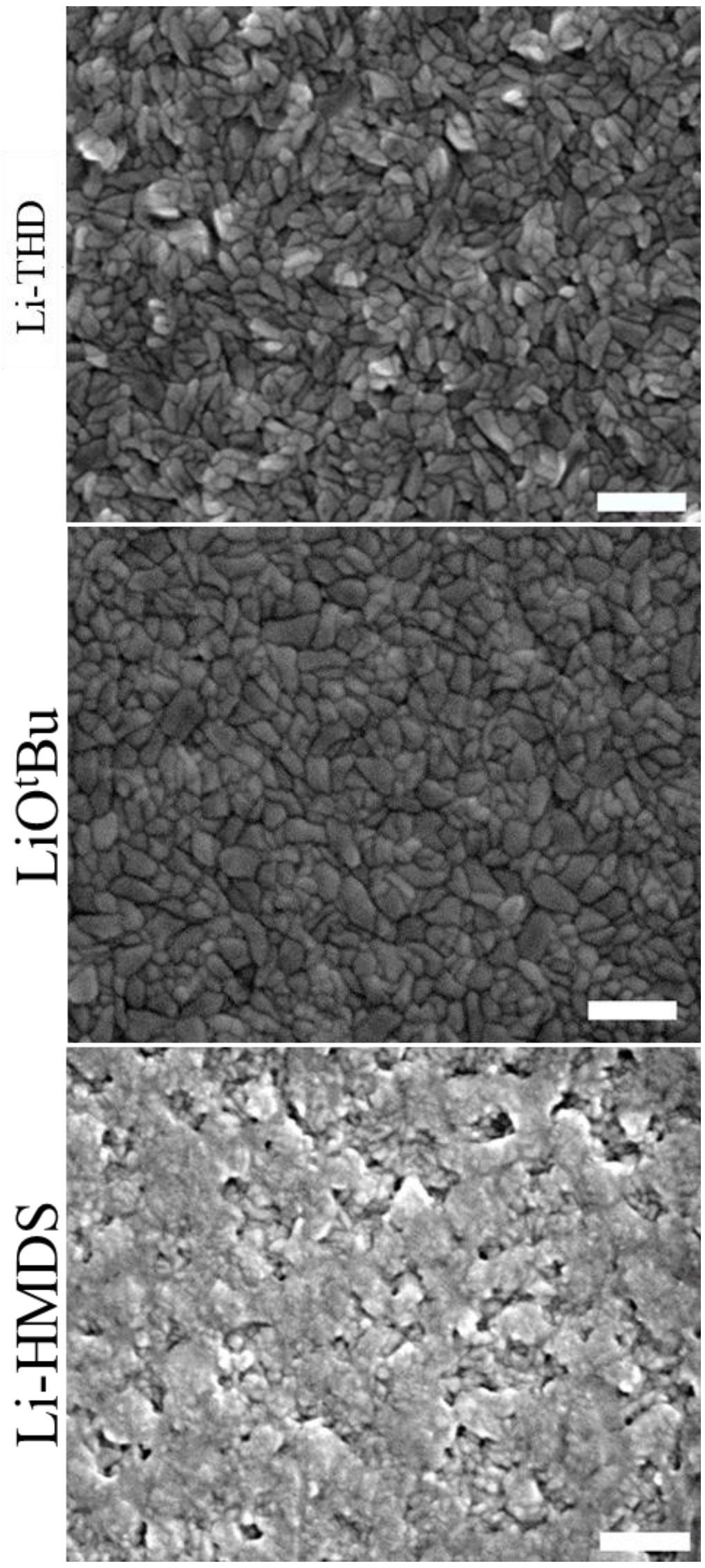

Figure S7. SEM images of Li-TPA deposited with 250 cycles of ALD/MLD. The lithium precursor used in the deposition is indicated on the left-hand side of the images. The scale bar in each image is 200 nm.

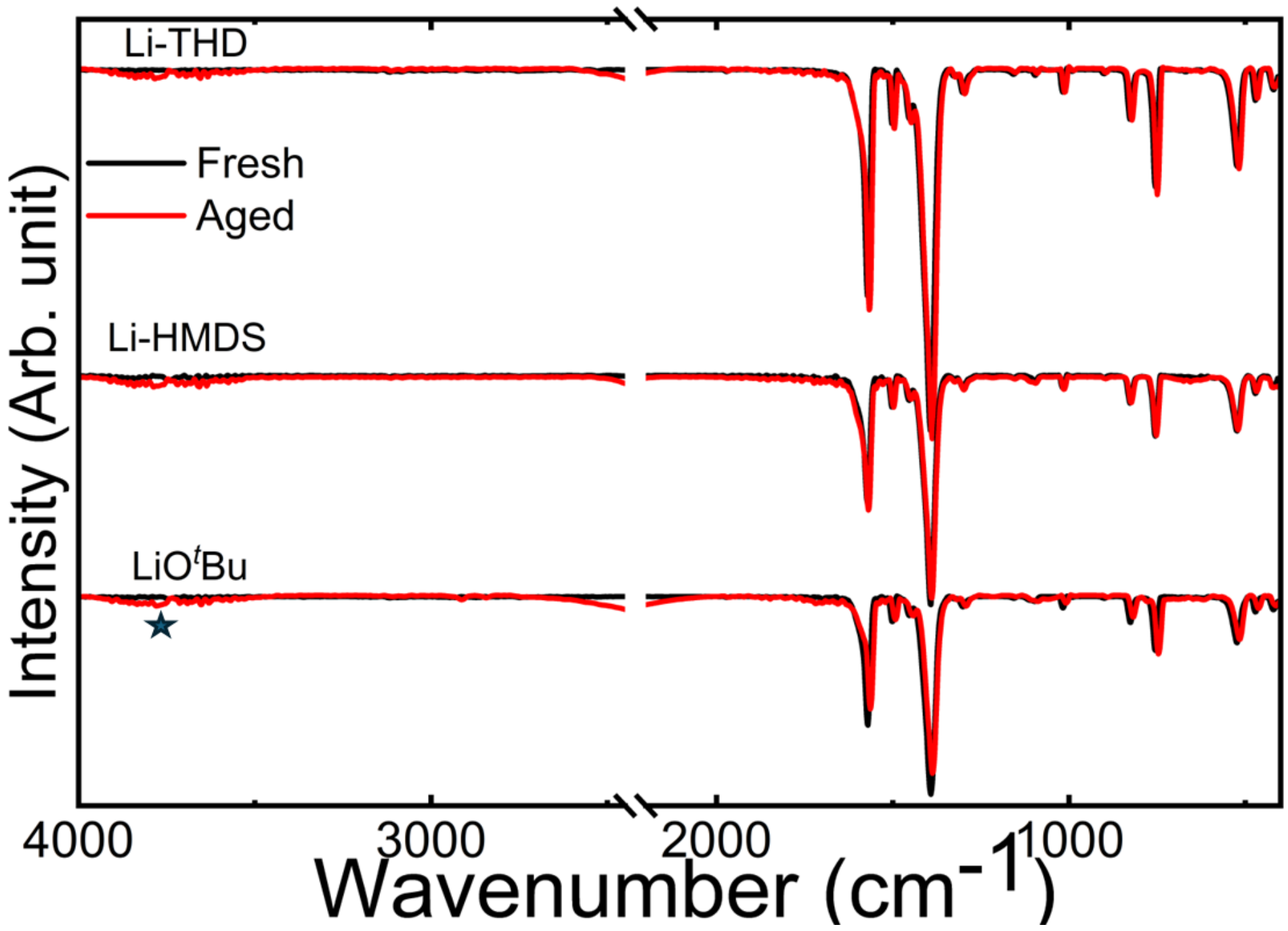

Figure S8. Comparison of FTIR spectra from fresh and one-year aged Li-TPA films. The bands marked with * are caused by adsorbed CO_2.

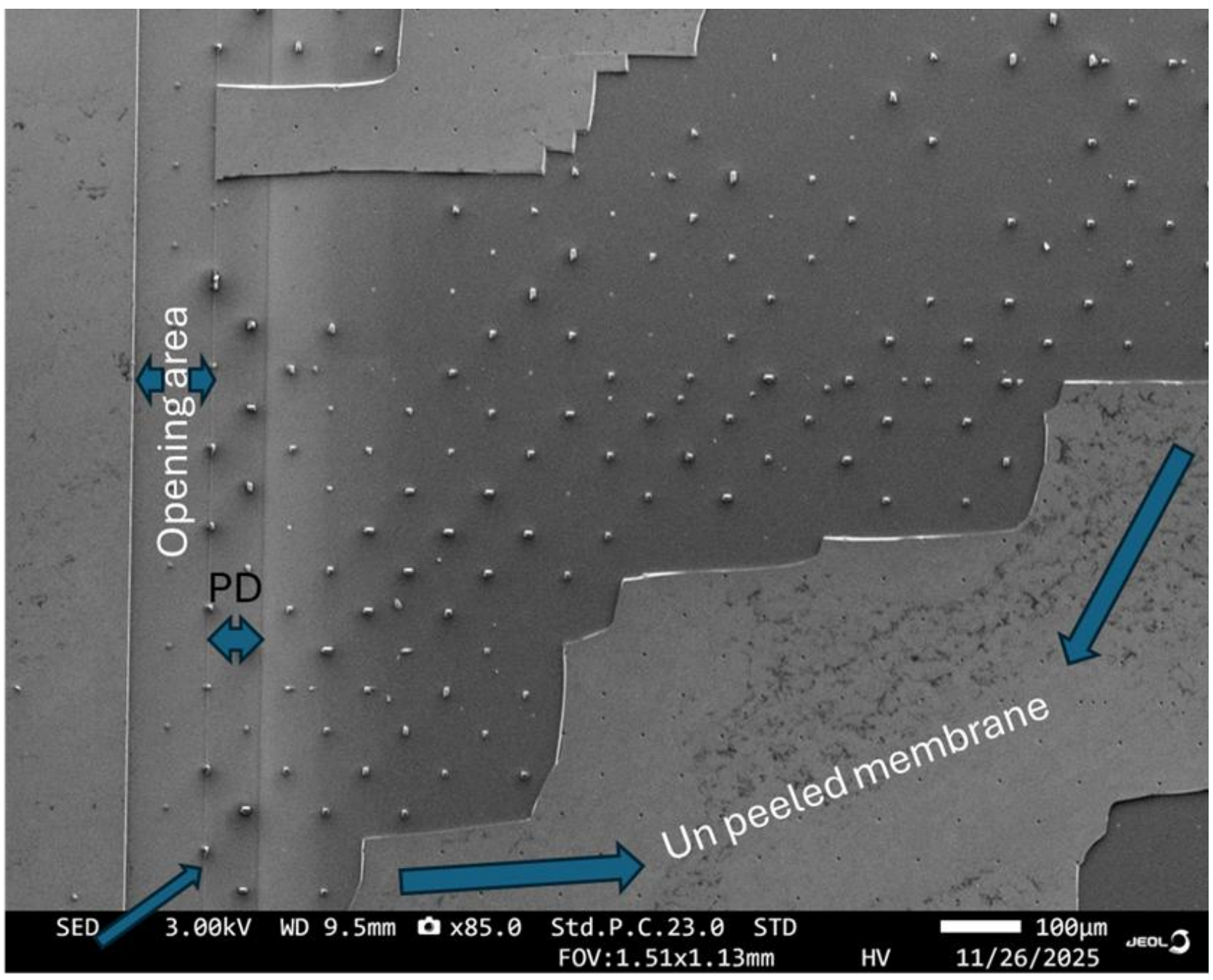

Cavity entrance

Figure S9. SEM image from PillarHall LHAR test structures after Li-TPA thin film deposition and peeling off the top-roof membrane. The top-roof membrane is not fully peeled off with adhesion tape approach, and the left-over membrane is indicated. Also indicated are the opening area (which is not covered with a membrane and acts like a planar silicon substrate), cavity entrance and penetration depth (PD, how deep the film deposits). Pillar residue is visible in the peeled-off area

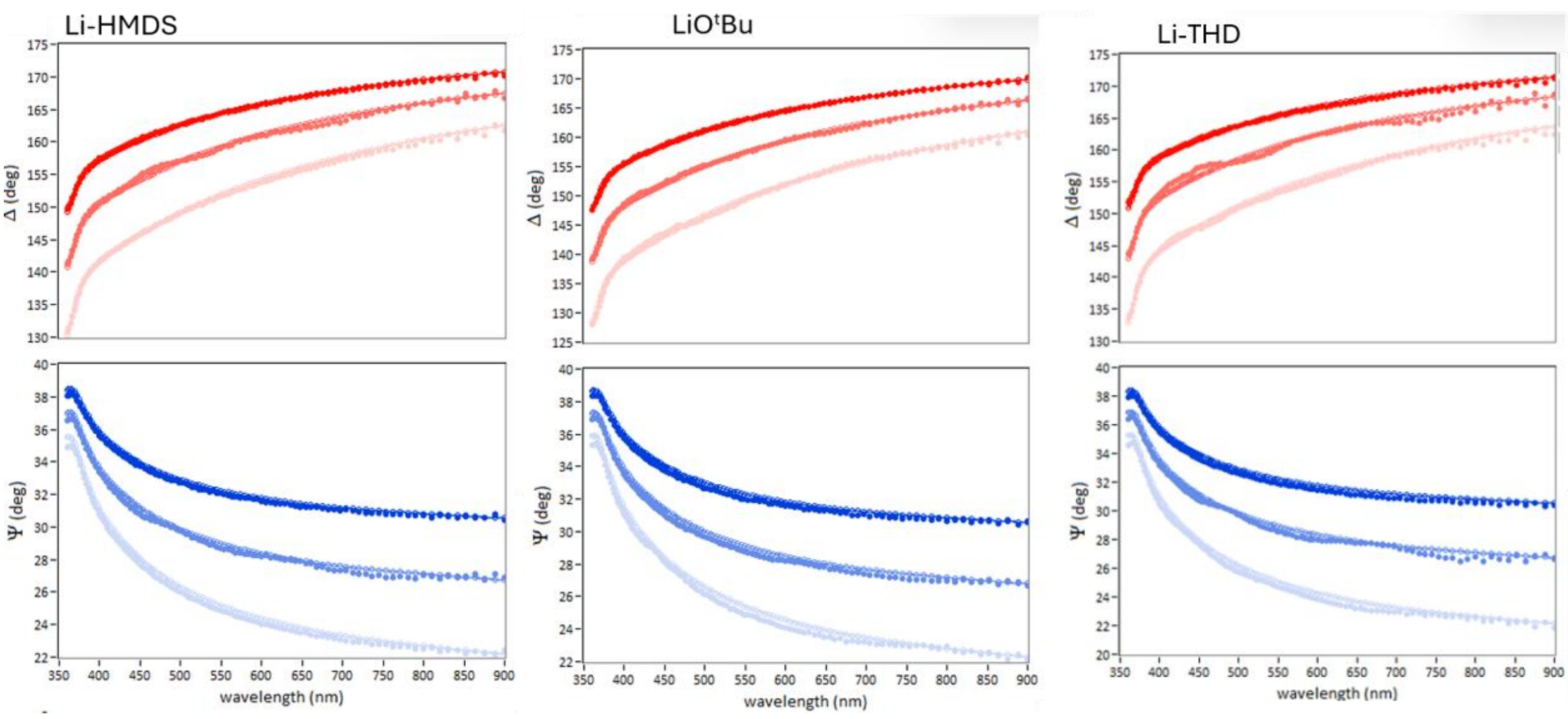

Figure S10. The thickness measurement fitting results for three different angles of incidence from Li-TPA thin films deposited in PillarHall samples. Hollow circles correspond to the fit, while solid circles correspond to the measured data. The lithium precursor corresponds to the Li-TPA measured indicated on the top of the images.

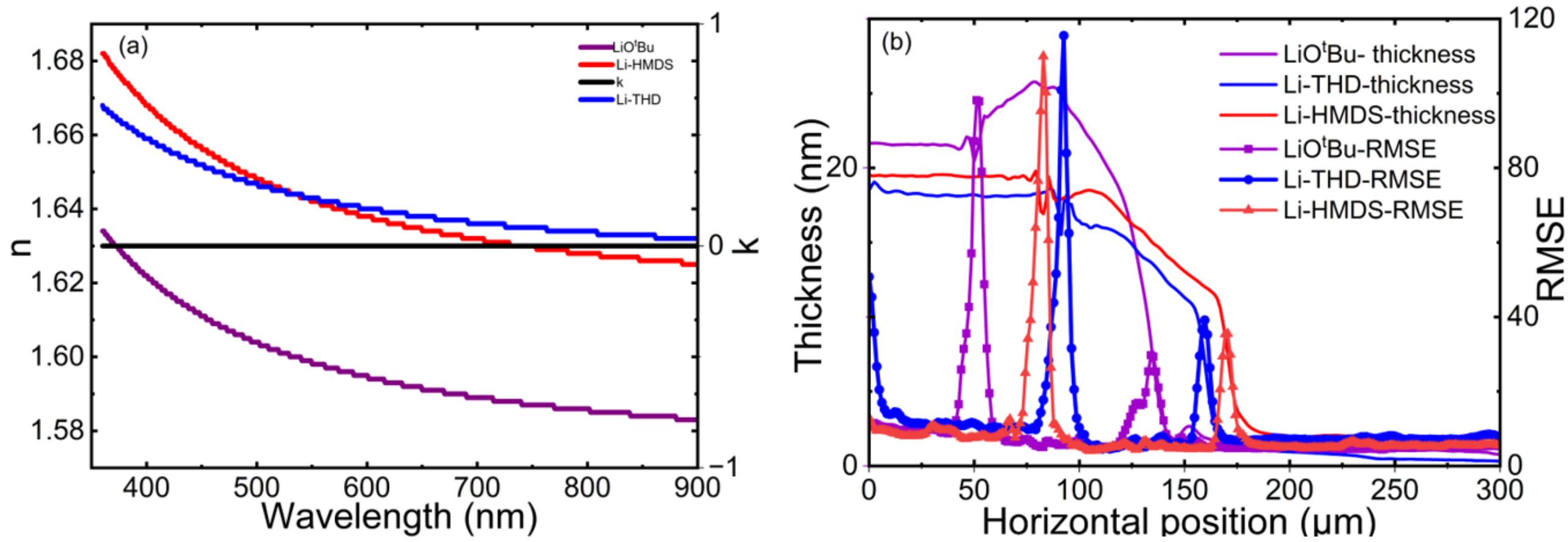

Figure S11. (a) Optical constants n and k obtained from ellipsometry measurements as a function of wavelength (nm). (b) Film thickness measured in the opening area and the cavity. The RMSE of the measurements is also shown.

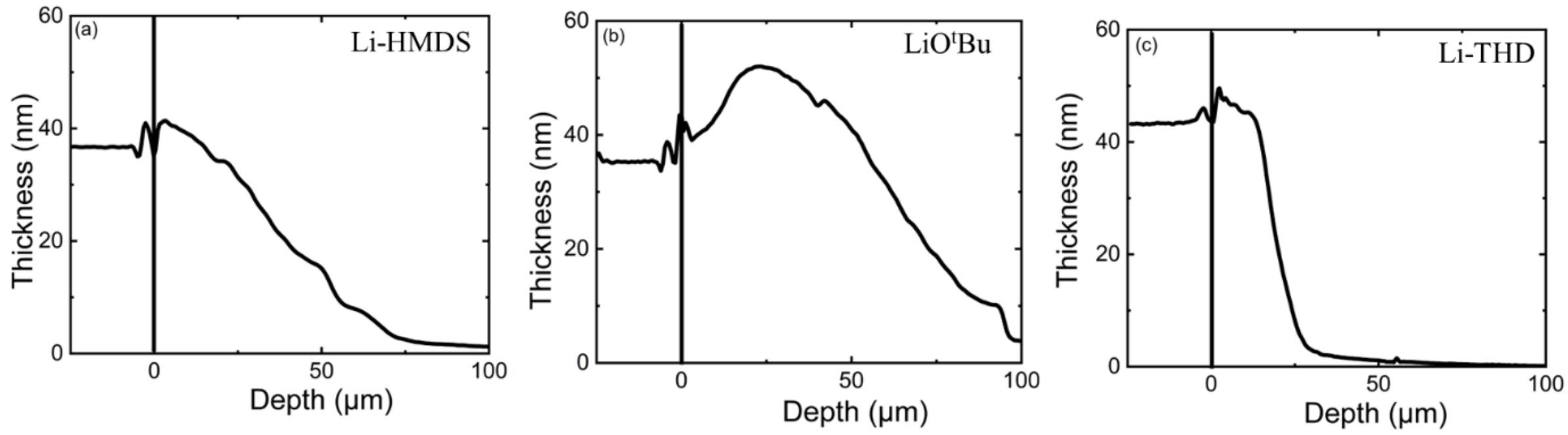

Figure S12. Imaging-ellipsometry-assisted thickness profiles for Li-TPA deposited on LHAR test structures with 100 ALD/MLD cycles using (a) Li-HMDS (5 s pulse), (b) LiO^{t}Bu (20 s pulse) and (c) Li-THD (5 s pulse). The deposition temperature used was 200 °C The lithium precursor used in each Li-TPA deposition is indicated in the corresponding thickness profile graph.

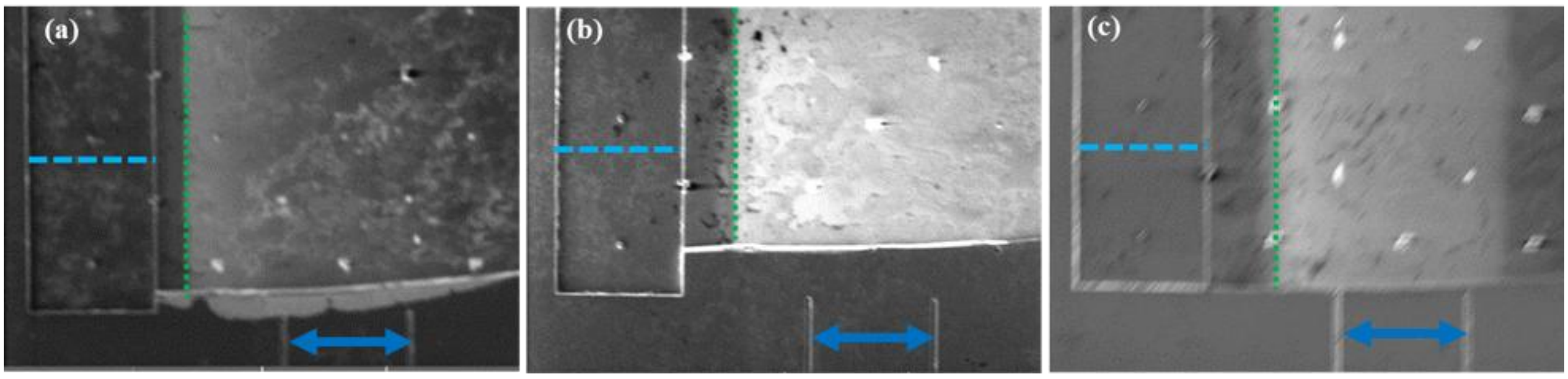

Figure S13. SEM images of Li-TPA thin films deposited with 100 cycles onto LHAR substrates using different Li precursors: (a) Li-THD, (b) LiO^{t}Bu, and (c) Li-HMDS. Following pulsing sequence was used: 5 s Li-precursor / 5 s N2 / 10 s TPA / 30 s N2. In each image, the opening area is indicated with a blue dashed line, the distance (100 µm) between two neighboring pillars with a double-sided arrow, and the film ending location (PD) with a green dotted line. The deposition temperature used was 200 °C.

References

1 S. Chapman and T. G. Cowling, The {Mathematical} theory of non-uniform gases: an account of the kinetic theory of viscosity, thermal conduction and diffusion in gases, Cambridge Univ. Pr, Cambridge, 3. ed., pr., 1999.

2 V. Cremers, R. L. Puurunen and J. Dendooven, Conformality in atomic layer deposition: Current status overview of analysis and modelling, Appl. Phys. Rev. 2019, **6**, 021302.
